# Zeolite in tissue engineering: Opportunities and challenges

**DOI:** 10.1002/mco2.5

**Published:** 2020-05-19

**Authors:** Payam Zarrintaj, Ghader Mahmodi, Saeed Manouchehri, Amin Hamed Mashhadzadeh, Mohsen Khodadadi, Morteza Servatan, Mohammad Reza Ganjali, Bruno Azambre, Seok‐Jhin Kim, Josh D Ramsey, Sajjad Habibzadeh, Mohammad Reza Saeb, Masoud Mozafari

**Affiliations:** ^1^ School of Chemical Engineering Oklahoma State University 420 Engineering North Stillwater OK USA; ^2^ Center of Excellence in Electrochemistry School of Chemistry, College of Science, University of Tehran Tehran Iran; ^3^ Polymer Engineering Department Faculty of Engineering, Urmia University Urmia Iran; ^4^ Biosensor Research Center Endocrinology and Metabolism Molecular‐Cellular Sciences Institute Tehran University of Medical Sciences Tehran Iran; ^5^ Université de Lorraine Laboratoire de Chimie et Physique‐Approche Multi‐Echelle des Milieux Complexes (LCP‐A2MC‐ EA n°4362) Institut Jean‐Barriol FR2843 CNRS Rue Victor Demange Saint‐Avold 57500 France; ^6^ Department of Chemical Engineering Amirkabir University of Technology (Tehran Polytechnic) Tehran Iran; ^7^ Department of Resin and Additive Institute for Color Science and Technology Tehran Iran; ^8^ Department of Tissue Engineering and Regenerative Medicine Faculty of Advanced Technologies in Medicine Iran University of Medical Sciences Tehran Iran

**Keywords:** biomaterials, porous materials, regenerative medicine, tissue engineering, zeolite

## Abstract

Tissue engineering and regenerative medicine follow a multidisciplinary attitude to the expansion and application of new materials for the treatment of different tissue defects. Typically, proper tissue regeneration is accomplished through concurrent biocompatibility and positive cellular activity. This can be resulted by the smart selection of platforms among bewildering arrays of structural possibilities with various porosity properties (ie, pore size, pore connectivity, etc). Among diverse porous structures, zeolite is known as a microporous tectosilicate that can potentially provide a biological microenvironment in tissue engineering applications. In addition, zeolite has been particularly appeared promising in wound dressing and bone‐ and tooth‐oriented scaffolds. The wide range of composition and hierarchical pore structure renders the zeolitic materials a unique character, particularly, for tissue engineering purposes. Despite such unique features, research on zeolitic platforms for tissue engineering has not been classically presented. In this review, we overview, classify, and categorize zeolitic platforms employed in biological and tissue engineering applications.

## 1 INTRODUCTION

Tissue engineering is an interdisciplinary under‐developing field in which the principles, standards, and capabilities of engineering, physics, and life sciences are integrated into a unique goal to identify, recognize, and eventually ameliorate the tissue functions. Providing the internal dysfunction organs with the opportunity of regeneration gifted by tissue engineering is the chief outcome of this progressively flourished field of science and engineering.^1^

The common strategies applied by the tissue engineers have undergone a flourishing era in the last decade by the progress in nanotechnology and nano‐biomaterials.^2^, ^3^ It has been accepted that a scaffold with a three‐dimensional (3D) porous structure provide appropriate substrate for cell migration and growth.^4^, ^5^, ^6^, ^7^ Annabi et al in a comprehensive survey highlighted the importance of controlling the porosity and the microarchitecture of hydrogels and overviewed the available techniques for a successful tissue engineering mission.^8^ Zeolite because of the crystallinity is a multiaspect material that has a micro‐ and nanostructure. Nanoporous and nanopatterned structures strongly affect the cells and tissue fate because high surface area, high protein adsorption, which improved the cell attachment, and specific topology guide the cellular activity toward specific purpose.^9^, ^10^ Moreover, it was claimed that the nanopore structure affects the cellular function by altering the conformation of certain cellular attachment proteins or by altering the surface energy.^11^, ^12^ Loh et al also emphasized the role of porosity and pore size as microenvironment for the successful incorporation of cells or growth factors in regenerating damaged tissues or organs.^8^, ^13^ It was shown that the preosteoblast cells attachment was enhanced on nanopore topography and in vivo results revealed that the nanoporosity enhanced colonization and cell diffusion within the scaffold.^9^ On the other hand, scaffolds should present adequate mechanical properties and stability to resist tensions and to keep the integrity of the engineered platforms.^14^, ^15^, ^16^ Such multiple requirements can hardly be responded at a reasonable cost by developing nanoporous 3D biomaterials with a nanosize structure. Several classes of minerals have already been examined in various biological applications. A unique family of multidisciplinary nanomaterials used in tissue engineering is zeolite‐based platforms that attracted continued attentions.

Thanks to their unique properties and exceptional stability in biological culture, zeolites were a versatile candidate for tissue engineering.^17^, ^18^ Zeolites are crystalline aluminosilicate materials constituted by an ordered array of micropores of dimensions about 0.4‐1.2 nm, close to those of many usual molecules. Owing to the ability of these materials to absorb water and release it upon heating, the term zeolite meant in ancient Greek “boiling stone.”

## 2 ZEOLITE SYNTHESIS AND PROPERTIES

### 2.1 Preparation method

Zeolite synthesis is a very significant area of research because zeolite with highly uniform microspores is very applicable in various industries such as catalysis, adsorption, and separation.^19^, ^20^ During the last 60 years, tons of zeolite with unique framework structure, compositions, and properties have been fully developed utilizing various synthetic methods. In the following paragraph, the various methods of zeolite preparation will be explained along with the useful parameters on the zeolite synthesis technique.^21^

#### 2.1.1 Hydrothermal synthetic method

Hydrothermal synthesis points out all the reactions in aqueous solutions that are happening in temperature and pressures more than 100°C and 1 bar, respectively.^22^, ^23^ Among existing methods for preparing the zeolite, the hydrothermal synthesis method is more reliable because of some unique advantages such as immense reactivity, facile control of solution or interface reaction, suitable condensed phase, and low energy consumption.^23^, ^24^ The hydrothermal synthesis based on reaction temperature can be considered as subcritical and supercritical synthetic reactions.^25^ In the subcritical technique, the temperature is between 100 and 240°C, whereas in the supercritical method, the temperature and pressure could increase up to 1000°C and 3000 bar, respectively. Supercritical synthesis can expedite the reaction rate, boost the hydrolyzation reaction, and immensely change the redox potential of the reactants.^25^, ^26^

Various factors impact the formation of a unique zeolite structure such as batch composition, reactant sources, Si/A1 ratio, alkalinity, water content, inorganic cations, organic templates, solvents, temperature, aging, stirring, and seeding.^25^

##### Bath composition

The bath composition of the reaction mixture determines the type of crystallized zeolite products. Like for instance, the bath composition could result in obtain zeolite structures such as Linde Type A (LTA), faujasite (FAU), analcime (ANA), and Sodalite (SOD).^27^

##### Si and Al sources

In zeolite structure, the major sources of silicon are sodium water glass, colloidal silica sol, fumed silica, tetramethyl orthosilicate (TMOS), and tetraethyl orthosilicate (TEOS).^28^ More importantly, the selection of the silicon source plays a vital role in the crystallization structure of the zeolite. In other words, the silicon source could also impact crystal size and the particle size distribution in synthesized zeolite.^29^ Because the silica source has an inevitable influence on the size and morphology of zeolite crystal, it is possible to control the zeolite crystal size of the zeolite by decent select of the silicon value utilized in the synthesis process. Based on the studies, less‐reactive silicon sources caused fewer nucleation sites and helped the formation of large crystals.^28^ Aluminum also has a significant impact on the crystallization formation of zeolites. The aluminum sources stem from materials utilized for zeolite synthetics such as sodium aluminate, pseudoboehmite, aluminum hydroxide, aluminum isopropoxide, aluminum nitrate, aluminum sulfate, and aluminum metal (Al powder or foil).^29^, ^30^

##### Si/Al ratio

The Si/Al ratio in the solution mixture has an inevitable effect in resolving the structure and stoichiometry of the prepared zeolite.^25^ For instance, zeolites with Si/Al ratio less than 5 like A (LTA), X (FAU), and hydroxy sodalite (SOD) are made from reaction mixtures with lower Si/Al ratio and strong alkalinity. However, zeolites with Si/Al ratio larger than 5 like zeolite beta (BEA), ZSM‐11 (MEL), and ZSM‐5 (MFI) are synthesized from mixtures with higher Si/Al ratio and weak alkalinity.^29^

##### Alkalinity

Fundamental Na20‐A1203‐SiO2‐H20 systems caused the crystallization of the zeolite structure. For the crystallization structure, alkalinity is determined as the OH‐/Si ratio or the H_2_O/Na_2_O ratio.^31^ Notably, higher alkalinity increases the solubility of the Si and Al sources, decreases the polymerization degree of the silicate anions, and expedites the polymerization of the polysilicate and aluminate anions.^32^ Therefore, enhancement of alkalinity will shrink the nucleation periods and make the crystallization formation faster. The alkalinity also can change the particle size of the zeolite, and even with increasing in alkalinity, particle size decreases consequently.^31^

##### Inorganic cations

Aluminum abundant zeolites are commonly synthesized under primary conditions by utilizing alkali‐metal hydroxide as the alkali source.^33^ The property of the inorganic cation is significant for the crystallization of zeolites. For instance, some zeolites are formed from aluminosilicate gel system that contains sodium components such as ANA, CAN, CHA, EMT, FAU (X), FAU(Y), FER, GIS, LTA, MAZ, MOR, MTT, MTW, MWW, and SOD; however, other zeolites such as EDI, KFI, LTL, MER, and TON were formed from crystallization structure in the presence of potassium‐containing species.^34^, ^35^

##### Solvents

The solvents are very significant for the crystallization of the zeolites. The solvents have an effect on their interaction with the structure‐directing agent (SDA) as the reaction species.^35^ The solvents also have an unavoidable impact on the size and morphology of the zeolite crystallization by their viscosity.^35^ Increase in the viscosity of solvent reduces both convective and diffusive mass transfer of reaction species. For the synthesis of zeolite (especially zeolites with large‐size crystal), a solvent with intermediate viscosity and hydrogen bonding is the preferred one.^35^

##### Crystallization temperature and time

All researchers give specific attention to the crystallization temperature because it has a strong impact on the formation of zeolite crystallization, such as the nucleation and crystal growth.^35^ An increase in temperature enhanced both the nucleation rate and the crystal growth.^36^ In other words, higher nucleation rates and the larger crystal growth rate were acquired at higher temperatures.^36^ The temperature may also change the morphology of crystals.^37^ Cundye et al showed that the length/width ratio of MFI zeolite crystals increased with temperature. It can be explained as various activation energies for the growth of each crystal structure. Another important parameter that should be considered is crystallization time. With increasing time, the crystallinity also increased.^38^ Davis et al studied the effect of time on crystallization, and they showed that with extended crystallization time, zeolite A (LTA) and zeolite X (FAU) dissolve to form zeolites sodalite (SOD) and P (GIS), respectively.^39^

##### Stirring

Several studies have exhibited that the crystal size of the zeolite could be modified by stirring.^40^, ^41^ For example, stirring creates smaller crystals because it expedites mass transfer, which accelerates supersaturation. Hanif et al showed that stirring could change the selectivity for the formation of various zeolite phases.^41^

#### 2.1.2 Solvothermal synthetic technique

In this synthesis technique, organic solvent or mixed organic water solvent were used to synthesize the zeolite instead of water in thermal technology.^42^ Xu et al prepared silicalite‐1 (MFI), ZSM‐39 (MTN), and ZSM‐48 utilizing alcohols rather than water. Generally, solvothermal synthetic technique reaction is slow in comparison with the thermal technique because the available solvating ability falls rather short of that of water.^43^ Solvents in the solvothermal synthetic technique show its effect on the crystallization of zeolites by their viscosity. Like for instance, higher viscosity of solvents reduces the mass transfer, which results in favoring the formation of large crystals.^44^ Ozin et al synthesized large single crystals of zeolite ferrierite (FER), silicalite (MFI), and the clathrasil dodecasil‐3C (MTN) by utilizing HF‐pyridine and HF‐alkyl amine solvents.^45^

#### 2.1.3 Ionothermal synthetic technique

In this method, ionic liquids and eutectic mixtures were used as solvent. Morris et al used this method to prepare zeolite. In this research, they used 1‐methyl‐3 ethylimidazolium bromide (m.p. 83°C) as both solvent and template in the synthesis of four different open‐framework for zeolite.^46^ Ionic solvents in this technique play as SDA and solvent. It also helps the crystallization process rate to be fast, low synthetic pressure, and high structure selectivity.^47^

#### 2.1.4 F^−^ synthetic technique

Flanigen et al reported the preparation of all‐silica materials in fluoride medium utilizing F^−^ than OH^−1^ as mineralizers.^48^ The mineralizing impact of F^−^ is usually used for silica‐based materials that are prepared in a solution with pH lower than 10‐11. An outstanding aspect of this method is the promotion of the growth of large single crystals. This occurs because of slower nucleation rate stems from lower supersaturation on the fluoride system than in the alkaline system.^49^, ^50^ Pang et al synthesized large single crystals of zeolites P (GIS), ZSM‐39 (MTN), Theta‐1 (TON), and ZSM‐5 (MFI) by adding HF or NH_4_F to the SiO_2_‐AI_2_O_3_‐SDA‐H_2_O system through utilizing various organic amines or ammonium cations as SDAs.^50^

#### 2.1.5 Microwave‐assisted hydrothermal synthetic route

Mobil Oil Corp., for the first time, used the microwave for zeolite synthesizing. Microwave‐assisted preparation is pretty faster, cleaner, and energy harvesting in comparison with conventional methods.^51^ Besides, the crystallization process had been immensely accelerated by using microwave radiation as a heating source. This technique has been utilized for the preparation of a number of zeolites such as zeolite A (LTA), faujasite (FAU), sodalite (SOD), analcime (ANA), beta (BEA), ZSM‐5 (MFI), silicalite‐1 (MFI), A1PO4‐5 (AFI), VPI‐5 (VFI), and cloverite (‐CLO).^52^

#### 2.1.6 Dry‐gel conversion synthetic

Dry‐gel conversion is a method in which crystalline zeolite is taken from dried aluminosilicate gel in a vapor. Matsukata et al categorized the dry‐gel conversion technique into two separate methods: (a) the vapor‐phase transport method in which a dry gel is crystallized in the vapor of water and volatile SDA and (b) the steam‐assisted crystallization (SAC) method in which a dry gel containing a nonvolatile SDA is crystallized in the steam.^53^ Crystallization by the SAC method might provide more information regarding structural changes in a parent gel, thus enabling a better understanding of crystallization mechanisms.^54^

### 2.2 Porosity and structure

Zeolitic frameworks are composed of TO_4_ tetrahedra [SiO_4_]^4−^ and [AlO_4_]^5−^ linked by oxygen atoms. Common building blocks of zeolite structures consist of three‐, four‐, five‐, and dix‐membered rings (*n*‐MR). Each *n*‐MR consists of *n*T atoms (T = Al or Si) linked in a ring by O ions and thus actually has 2*n* atoms (eg, a 6‐MR unit contains 12 total atoms). These building blocks are arranged in the different zeolitic frameworks to form larger rings, which represent the molecular pores. Depending on the peculiar arrangement of the elemental building blocks, channels and cages of different sizes and connectivity could be present. Eight‐, 10‐ and 12‐MR are found in many usual zeolitic structures and are commonly referred to small, intermediate, and large pores, respectively. This feature is at the origin of the well‐known porosity of zeolites, with specific surface area (from N_2_ sorptiometry) usually in the range 300‐900 m^2^/g. The presence of pores of uniform and definite sizes allows mixtures of molecules of differing structures to be separated and explains why zeolites belong to the class of molecular sieves.^55^, ^56^

The different structures of zeolites could be distinguished from the pore size, the zeolite dimensionality/pore connectivity type (1D, 2D, and 3D), and the orientation of cages, cavities, and windows. Although more than 200 different zeolitic structures are nowadays described in the databases provided by the International Zeolite Association (IZA), the zeolitic structures with wide availability and application are probably less than a dozen. The labeling of a zeolitic structure is defined by a three‐letter code, which could differ from the commercial/usual designation. Natural zeolites are generally formed in alkaline environments from volcanic sediments and materials and are found in different structures, such as stilbite, clinoptilolite, erionite, anacime, mordenite, and so forth. Besides being rather expensive, natural zeolites often have defaults and irregularities in their structures, as well as different chemical compositions, depending on their origin. Hence, it is the development of laboratory methods of synthesizing zeolites that led to the many commercial applications of zeolites. From the commercial viewpoint, the most important zeolite sales are related to the FAU, Linde Type A (LTA), and MFI structures. For instance, the faujasite framework consists of sodalite cages that are connected through hexagonal prisms. The pores are arranged perpendicular to each other. The pore, which is formed by a 12‐membered ring, has a relatively large diameter of 7.4 Å. The inner cavity has a diameter of 12 Å (supercage) and is surrounded by 10 sodalite cages. In faujasites, adsorption mostly occurs in supercages or at the windows pointing toward them. Being very schematic, zeolitic structures could be classified into three categories: small‐pore zeolites with pore diameter from 3 to 4.5 Å with LTA structure as representative (3A and 4A zeolite). Such zeolites are able to adsorb small molecules including water, diatomic gases, ammonia, and *n*‐paraffins; medium‐pore zeolites with pore diameter between 4.5 and 6 Å such as in MFI (ZSM‐5) or ferrierite (FER) structures, which could accommodate the presence of small aromatics or branched alkanes; large‐pore zeolites with pore diameter above 6 Å such as faujasites (FAU) and mordenites (MOR), which allow the diffusion of highly‐branched paraffins and more bulky molecules in their pore system.^57^, ^58^

### 2.3 Chemical properties

In each zeolitic structure, the substitution of the tetravalent Si by trivalent Al leads to a negative charge, which is compensated by the presence of cation M, of *n* valence (so‐called charge‐compensating or extra‐framework cation):

$$M_{x/n}{\left[\mathrm{Al}O_{2}\right]}_{x}{\left[\mathrm{Si}O_{2}\right]}_{y}.zH_{2}O$$

where *x* *+ y* represents the total amount of tetrahedra per lattice unit; *y*/*x* represents the molar ratio Si/Al. This ratio has to be higher than 1 (*Lowenstein rule*) and could be even close to infinite (in silicalite and zeosil structures).^59^

Some structural types can be synthesized through a rather high range of Si/Al ratio (eg, faujasites using dealumination processes), whereas others are stable only under a restricted domain. After hydrothermal synthesis processes at 100‐200°C (mostly under alkaline conditions in presence of organic templates), the charge‐compensating cations are often of the Na^+^ and NH_4_^+^ types (K^+^) and maintain the electroneutrality of the framework. Hence, commercial synthetic zeolites are often found in sodium or ammonium forms. Nevertheless, the possibility to exchange these cations easily by other monovalent or multivalent cations (eg, H^+^, Ca^2+^, Ag^+^, and Cu^2+^) is at the origin of the well‐known cation‐exchange properties of zeolites. These properties can be used for the retention of cations from liquids and solutions, or even to incorporate specific metallic species in order to confer to the zeolite‐specific adsorption or catalytic properties.^60^ For instance, the presence of protons on exchange sites is used to obtain acid catalysis properties, whereas the exchange with transition metal cations is used in oxidation/reduction catalysis or to get specific trapping sites. By calcination of the ammonium zeolite form or exchange with protons, hydroxyl groups of the Si(OH)Al type are generated. They are considered as Brönsted acid sites (BAS), because they can protonate Brönsted bases.^61^ Their concentration decreases as the Si/Al increases, but their specific acid strength increases reversely. Zeolitic acidity is much stronger than that formed in amorphous aluminosilicates, which is usually based on the Al‐OH group. Moreover, the acid strength of BAS is known to strongly vary with the structure (MOR > MFI > BEA > FAU). The second type of sites is represented by Lewis acid sites (LAS). These sites are extra‐framework aluminum (defects) or a cation on a charge‐compensating site bearing dangling bonds (Ag^+^, Cu^2+^/Cu^+^, etc). By contrast, zeolites exchanged with alkali metals have mostly basic character. Many parameters are influent on the number of cations, which could be incorporated by ionic exchange in order to achieve specific adsorption properties. These parameters are mostly the Si/Al ratio (which imposes the theoretical exchange capacity), but also the nature, size, and charge of the cation, the temperature, the pH, and the exchange time.^62^

On the other hand, the adequate control of the Si/Al ratio (when possible) allows also to finely tune the hydrophilic/hydrophobic character of a given zeolite. The hydrophilic character increases as the Si/Al ratio in each zeolitic structure decreases. Zeolites with low Si/Al ratio are very good drying agents, even at low partial pressure of water. Hydrophilic centers are OH groups (silanol) or cations associated with tetrahedrally coordinated aluminum. By contrast, Si‐O‐Si bonds of the framework are homopolar. This explains the highly hydrophobic character and the more important hydrothermal stability of almost pure‐silica zeolites (with high Si/Al ratio).^63^

### 2.4 Physical aspects

As far as interfacial aspects are concerned, physical properties of zeolites are also important in determining the properties of these materials. Zeolites, being inorganic aluminosilicate materials, are rather robust and can withstand elevated temperatures and gamma‐irradiation without damage to their crystalline structure. Nevertheless, zeolites with low Si/Al ratio may degrade and loose crystallinity at high temperatures in the presence of liquid water. Acidic medium also promotes dealumination of the framework, but advantage could be taken from this reactivity in order to produce zeolites with higher Si/Al ratio (or alternatively by steam treatment at high temperature).^64^

The zeolite crystal size is also important in determining the performances of zeolitic materials for a variety of applications. Depending on synthesis conditions, the crystal size of zeolites could be very large, from hundreds of micrometers, or very small, in the nanometric range. Nevertheless, the average size, approximately 1‐3 µm, is by far above the sizes of micropores exhibiting a molecular‐sieving effect. Hence, and due to limitations of mass transfer, the diffusion rates of adsorbates or reactant molecules within the micropores located within zeolite crystals are lower than the intrinsic adsorption/reaction rates.^65^ By contrast, nanocrystalline zeolites have larger external surface areas and therefore exhibit much less diffusional problems, because the distance from the surface to the bulk of the particles is extremely reduced. In order to obtain nanocrystalline zeolites, the addition of a surfactant into synthetic solutions of zeolites is a promising method to control the crystal size and improve the crystallinity. Moreover, the advantages related to nanometric size of zeolite crystals can be combined in hierarchical zeolites by adding secondary porosity (at meso‐ and macroscale) superimposed on the primary microporous structure. By comparison with classical zeolites, hierarchical zeolites still exhibit molecular sieving ability while also providing fast mass transfer, and this feature is important in many adsorption and catalytic applications. For instance, the secondary porosity was often found to increase the lifetime of zeolitic catalysts by limiting deactivation processes while also providing the possibility to control the deposition of active phases on the external surface.^66^, ^67^

Zeolites can also be obtained under the form of coatings and films as barriers or alternative to surface passivation treatments. By having the capacity to host extra ions and molecules in their regular porous structures, zeolite coatings and films can react to external stimuli from environment or act as corrosion inhibitors. In situ crystallization and sol‐gel coating are the most widespread methods to prepare them. By the in situ crystallization method, direct synthesis of zeolite crystals on a metal support could be achieved, allowing optimal zeolite/metal adhesion. By contrast, the sol‐gel technique is easier to scaled up, and it is also possible to obtain adhesive and cohesive coatings.^68^

Overall, the zeolite identity and properties are defined by many factors, primarily the structural type, which imposes the zeolite dimensionality, the micropore size, and pore connectivity. In addition, chemical factors such as the Si/Al ratio and the heteroatom content and location, as well as physical properties related to the crystallite size and zeolite architecture, are of great importance in many applications. Most zeolitic structures are noncytotoxic (except erionite), biocompatible, and have high mechanical strength, making them suitable components to be incorporated in composites or scaffolds for biomedical applications. By selective functionalization of their external surface, it is possible to increase their adhesion to organic/biopolymer materials.^69^, ^70^

The main characteristic that makes zeolites a promising candidate for tissue engineering applications in comparison with the other porous materials is the diversity and heterogeneity of their pore sizes and 3D shapes. The porosity network of zeolite with different pore size from 2 to 50 nm, limited by the framework, contains holes and connecting cavities of similar size, which can perform on toxicants, interact with them, and introduce vital minerals into the body using ion‐exchange or carrying within structure. Zeolite enables to adsorb pollutants, bacteria, and viruses, which can be used as a body detoxification. Moreover, due to the hydration effect, zeolite interact with water, which is beneficial for skin, and such feature can be utilized to improve the function of the connective tissue and prevent wrinkle forming. Zeolite stimulates the cell growth, which increases the healing and regeneration process and protects electrolytes and electrophysiological procedures. Zeolite has a solid advantage including decreasing the side‐effect of the drugs, detoxifying due to the ion‐exchange feature, versatility of formulation such as conjugating to drug, DNA, antibodies, carbohydrates, and peptides due to the wide range of porosity and different type of structure, reducing the inflammation, destroying the tumor cells due to the electrical, optical, and magnetic feature, and assisting the blood clotting.^71^, ^72^

**FIGURE 1:**
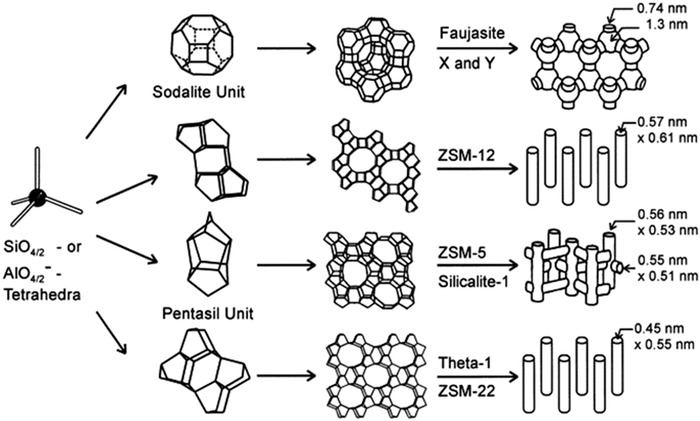
Different structural forms of zeolites identified so far. [Zeolite Socony Mobil (ZSM)]. Adopted with permission^75^

Moreover, zeolites are active biomaterials identified as biocompatible, antibacterial, nontoxic, and highly porous structures.^73^ Figure 1 shows that zeolite pores have sizes ranging from large empty spaces, or super‐cages within their configuration to tiny microporous holes or interconnected rod‐shaped hallow channels, which can host large positively charged moieties, such as Na^+^, K^+^, Br^+^, and Ca^2+^, or subnanometric chemicals and cell substances and even proportionally large molecules and active groups, including water, ammonia, carbonate, and nitrate ions. Furthermore, the basic structure of zeolite is biologically neutral and mostly has zero toxicity for tissues.^74^

**FIGURE 2:**
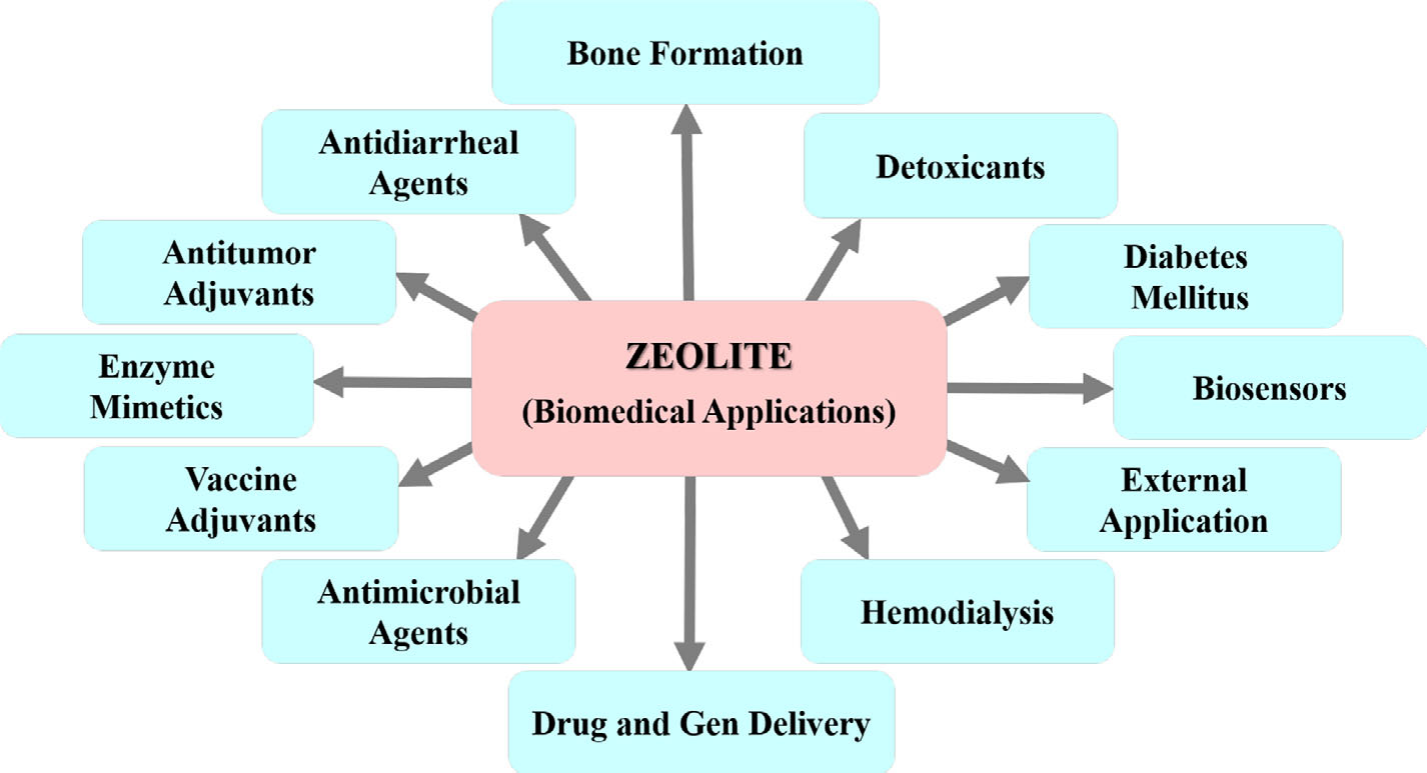
A summary of the zeolite nanocrystals used in biomedical applications. Zeolites have positive effect on detoxification of animal and human living organisms, the treatment of diabetes mellitus and hemodialysis, construction of biosensors and biomarker detection of diseases, controlled drug and gen delivery, external applications, enzyme mimicry, and bone formation. Besides, zeolites used effectively as vaccine adjuvant, antimicrobial, antitumor, and antidiarrheal drugs

The bioactivity, biocompatibility, and nontoxicity of nanocrystalline zeolitic structures gives them a great potential for medical and biological applications. The prevalent use of zeolites in veterinary and human medicine has been addressed in several reports.^73^, ^74^, ^76^ Nevertheless, no comprehensive classification has ever been made to understand the worth of this versatile microporous framework for tissue engineering uses. A summary of the zeolite applications in biomedicine field is represented in Figure 2.

In this review, applications of zeolite‐based structures in tissue engineering are summarized. Moreover, the effects of zeolitic frameworks on the biological, mechanical, chemical, and morphological properties of tissue engineering‐oriented platforms have been discussed from wound dressings, hemodialysis, scaffolds, antimicrobial agent materials, implants and coatings, removal of toxic materials and delivery of nutrients, contrast agent, and dental application perspectives.

## 3 INTERACTION OF ZEOLITE WITH CELLS

Materials selection for tissue engineering and regenerative medicine applications is a challenging issue. The interaction of substrate with the cells plays the key role in tissue regeneration, which is related to the substrate chemical (atom ratio, Brönsted's and Lewis's acidity, and hydrophobic/hydrophilic properties) and physical properties (structure, porosity, crystallinity, and topology).*^77^* Interaction of the protein‐zeolite determines the cultured‐cells fate because the secreted protein by cells can enhance the cellular attachment, adhesion, growth, and activity. Tavolaro et al evaluate the protein adsorption on various types of zeolite. It was noticed that the zeolite structure, chemical composition, morphology and size of the crystals, the Brönsted acidity, the number and the distribution of defect, hydroxyl groups (which its acidity can be enhanced by presence of the strong Lewis center [Al^3+^] or decreased by using fluoride ions), protein incubation time, and temperature affect its interaction with protein.^78^ Using acid site (acid Brönsted sites) on zeolite crystalline surface, zeolite converts the basic molecules to conjugated acid; hence, by acid silanol groups adjusting the protein immobilization can be controlled. Moreover, “IN and NO” crystal type exhibited higher protein adsorption than seeded crystallization.^79^ It was reported that the protein adsorption enhances near the protein isoelectric point (pI) value. Point of zero charge (PZC) of zeolite has strong effect on its protein adsorption ability to determine the pH of the immersed zeolite surface with zero net charge and the pH resulting in equivalent concentrations of positive and negative groups derived from the hydrolysis products of material dissolved from the crystal. It should be mentioned that the pH increase above the pI value results in protein adsorption because of enhancing the inorganic substrate acidity.^80^

Human cell growth outside the body is a harbinger of successful therapeutic method; therefore, designing proper substrate is a prerequisite need for tissue engineering.^81^, ^82^ Zeolites can be utilized as a host substrate for cellular adhesion with modulated surface thanks to their silanol groups amenable for cell adhesion and growth. Properties of zeolite can be easily manipulated because of their high porosity with adjustable pore size, wide ranges of crystal size as an anchor for cell adhesion, adjustable acidity using Si/Al ratio, or introducing metal ions within the crystalline network.

**FIGURE 3:**
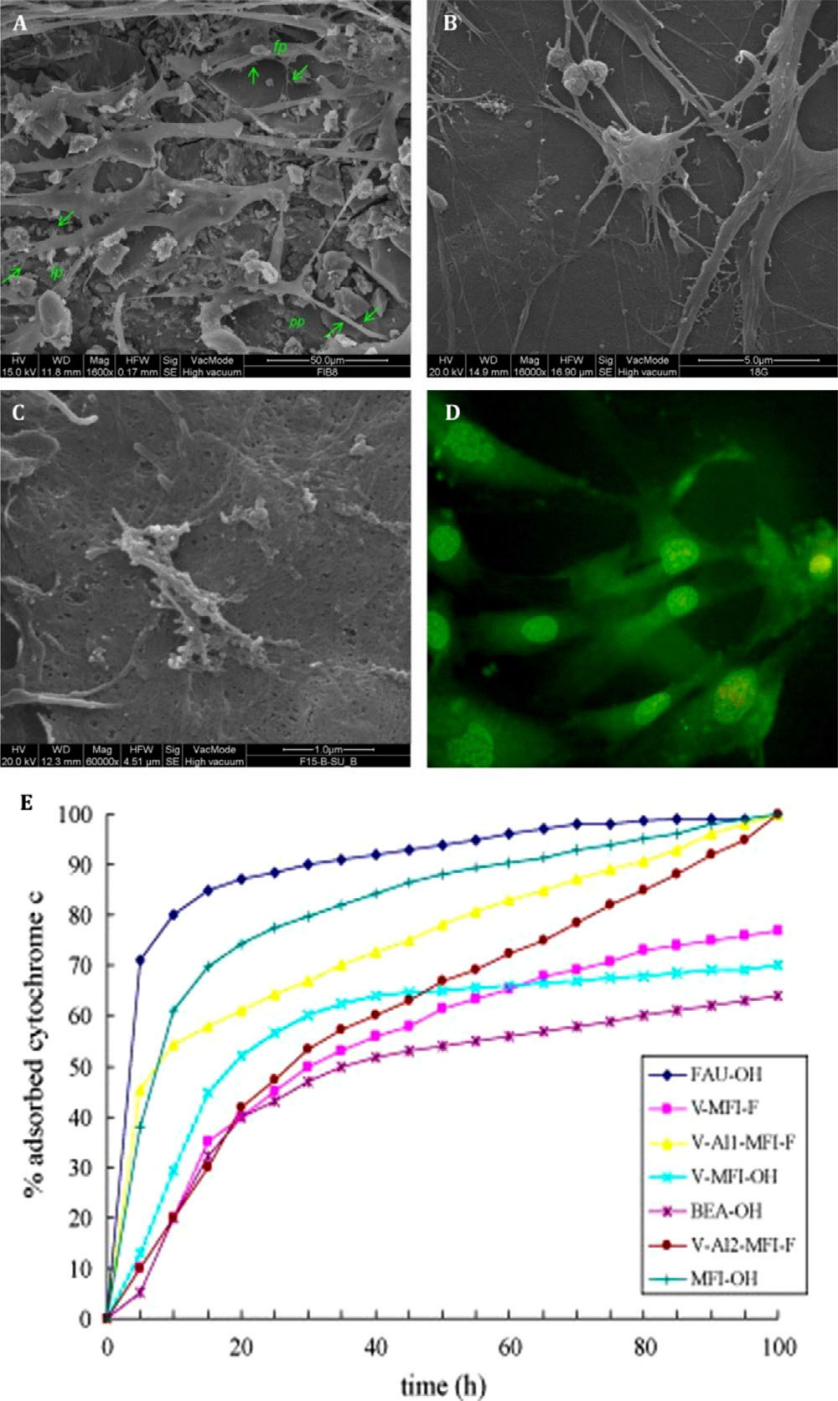
FESEM microphotograph of human fetal fibroblasts grown on FAU zeolite membrane. The cell bodies are less adherence to scaffold, and stretch out very robust arms, which spread in all direction and anchor cells to the inorganic support breaking its surface. It is possible to observe the presence of several pseudopodia (pp), filopodia (fp), and lamellipodia (lp). B, FESEM microphotograph of the MOR zeolite membrane surface covered with the adhered fibroblast cells. It is evident the presence of various filopodia having growth cones and lamellipodia with numerous ruffles. C, FESEM microphotograph of fibroblasts incubated using the MFI zeolite membrane after five culture days. D, Fluorescence microscopy image of fibroblasts incubated using the MOR zeolite membrane after three culture days (Acridine orange). E, Adsorption isotherms of cytochrome c on different zeolite crystals synthesized (adsorption experiments are carried out at pH 7.8). [Faujasite (FAU), vanadium silicalite‐1 (V‐MFI‐OH), zeolite β (BEA‐OH), silicalite‐1 (MFI‐OH), V‐Al2‐MFI‐F (vanadium ZSM‐5), vanadium silicalite‐1 (V‐MFI‐F)]. Adopted with permission^83^

Tavolaro et al evaluated various types of zeolites, including Mordenite (MOR), Zeolite Y (FAU), Zeolite β (BEA), Silicalite‐2(MEL), Silicalite‐1 (MFI), and V‐Silicalite‐1 (MFI), as substrate for cell growth.^83^ All studied structures exhibited significant growth with respect to tissue culture polystyrene (TCPS) as a reference. Among the investigated various type of zeolite, MEL exhibited the highest cell viability because of the nonflat and jagged nanocrystals, which induced cells and extended more filopodia to form focal adhesion. Fibroblast cell morphology on TCPS and MFI tended to spherical shape, whereas cell morphology on MEL and MOR tended to spindle shape. Cells exhibited high number of pseudopodia and filopodia to make a connection with juxtaposing cells. It was deduced that the intercrystalline spaces and cavities of the zeolites were behind the enhanced adhesion and morphology of cells (Figure 3). Cytochromes as carrier proteins contain heme that play an essential role in electron transfer and adenosine triphosphate (ATP) formation to be used as a model for biochemical redox reaction. Immobilization of cytochrome within the zeolite framework preserves properties for biotechnological applications.^84^

**FIGURE 4:**
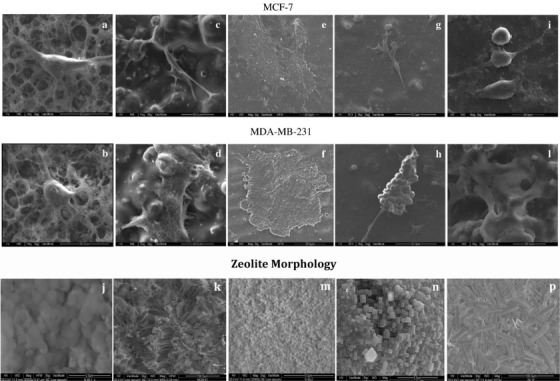
Cell morphology on zeolite. SEM image of MCF‐7 and MDA‐MB‐231 adhered on the same type of scaffold: (A) and (B) on N‐Sil‐1 (I); (C) and (D) on MCM‐41 (K); (E) and (F) on G‐Sil‐2 (M); (G) and (H) on LTL (N); (I) and (L) on ZeoA (P)^86^

Tavolaro et al examined the response of two different human breast cancer cells, MCF‐7 and MDAMB‐231, with different growth rate, response to hormones, invasiveness, and metastatic activity inside the various types of zeolitic substrates. By interacting with cancer cells using silanol groups, zeolite affected the cellular morphology. MCF‐7 morphology exhibited distinct, thin, and elongated cell bodies with the presence of many long filopodia well anchored to the rougher parts of the membrane (crystalline agglomerates). On the other hand, MDA‐MB‐231 cells exhibited an enlarged polymorphic spots fused together with cell bodies adhering in a homogeneous manner on the membrane (Figure 4).^85^, ^86^ Initial cell tethering and filopodia exploration is accompanied by lamellipodia ruffling, membrane activity, and cell spreading. Matrix assembly sites generate on the ventral plasma biological membrane by the endogenous matrix secretion. Using integrin recruitment, cell‐matrix contacts formed anchoring focal complexes at the lamellipodium leading edge, which were reinforced intracellularly to form stronger focal adhesion plaques upon enhanced intracellular and/or extracellular tension. Therefore, it was explored that zeolite surface properties could control the cellular activity and behavior.^87^

Adsorption of DOX and 5‐MOP on zeolite membrane scaffolds was also investigated. After 6 h of incubation, the pure zeolite membrane presented better adsorption behavior by 90%. Moreover, rapid adsorption of DOX emerging for MCF‐7 cells on the hybrid membranes took place, which increased to 50% in the next 24 h of incubation. Likewise, the MDA‐MB‐231 cells located on the same scaffolds displayed that DOX was absorbed closely to the MCF‐7 condition with a slimly lower rate, suggesting the same manner regarding the type of membrane.^85^ These tests were performed under similar condition for 5‐MOF and the first analyses on data indicated a better adsorption for MCF‐7 scaffolds in comparison with MDA‐MB‐231 cells. The overall adsorption results verified that hybrid zeolite adsorption capacity was lower than pure zeolite scaffolds. Analysis of cell viability showed that the pure zeolite offers a better viability and a higher negative gradient following the antineoplastic drug encounter than the hybrid zeolite membranes.^85^

**FIGURE 5:**
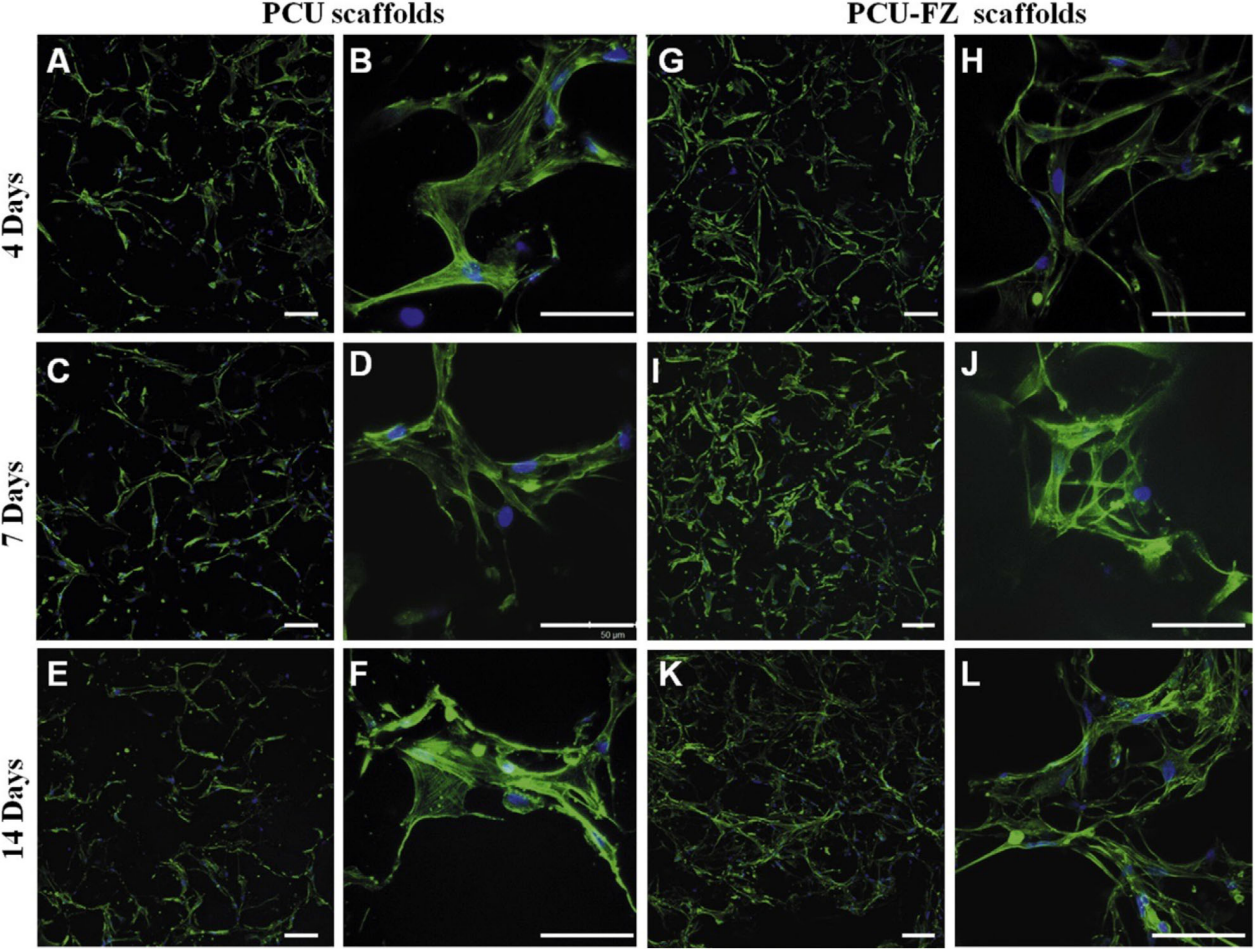
Confocal microscopy images of HCASM cultured on PCU and PCU‐FZ scaffolds for (A, B, G, and H) 4 days, (C, D, I, and J) 7 days, and (E, F, K, and L) 14 days. HCASMC attached to both PCU and PCU‐FZ scaffolds had similar morphologies but the cell density appeared to be higher on the PCU‐FZ scaffolds. Adopted with permission^88^

Oxygen supply of tissue is one of the most crucial parameters in tissue regeneration. Seifu et al evaluated oxygen transport rate of zeolite‐based nanomaterials. Fluorinated zeolite samples were placed in a 3D polyurethane structure as an oxygen vector system.^88^ Although zeolite had no significant effect on the porosity of scaffold, nor pore size and morphology, cell penetration properties for zeolite‐incorporated samples were approximately two times more than the reference sample.^88^ Fluorinated molecules founded to be efficient in dissolving oxygen, because they were mostly located on the surface of the zeolite particles, which were in direct contact with the bulk fluid subsequently. These results on the other hand revealed no similarity with the pure fluorinated emulsions behavior due to the fact that the oxygen molecule was confined in the main central section of the particle. Accordingly, the sensor calculated the total dissolved oxygen rates in the culture media. Considering this phenomena, the improved oxygen concentration could be related to the presence of perfluorodecyltriethoxysilane (PTES) at the surface of the zeolite layers. Gaseous phase was delivered by perfluorinated complexes because of increase in the solubility degree in agreement with Henry's law. Moreover, this enhancement was associated with the presence of loose, nondirectional van der Waals interactions, which resulted in low cohesive energy and easy mutual solubilization of oxygen in the fluorine section of the composite connected to the surface layers present in the scaffold.^88^ Low intermolecular interactions and the low polarizing power of fluorine converted into van der Waals bounds within a zeolitic monolayer. In the procedures where oxygen molecules were transferred with hemoglobin assistance, the rate of oxygen release was modulated by the hemoglobin as oxygen molecules were connected to the molecules. However, in oxygen‐incorporated zeolite compounds, they could be released whenever needed in tissue media that clearly showed the zeolite benefit for better cellular oxygen supply (Figure 5).^88^

## 4 ZEOLITE IN TISSUE ENGINEERING AND REGENERATIVE MEDICINE

### 4.1 Skin tissue engineering/wound healing

Skin as largest organ in the body plays an important role to protect the body against bacteria and disease attack. During the skin injury, the body endeavor to heal the wound and regenerate the skin. In some cases, such as a large wound and diabetic wound, the body cannot regenerate the skin lonely and need some assistance. Wound dressing is common substrate that is used for wound healing. Based on the wound (surgical wound, burning wound, and diabetic), the proper substrate is selected to heal wound. Zeolite because of its unique properties can play an essential role in wound dressing.^89^, ^90^, ^91^

Ninan et al synthesized gelatin/hyaluronic acid (HA)/faujasite porous scaffolds with low surface energy as a wound dressing using freeze‐drier. Zeolite binding to the main polymer resulted in pore size reduction, which benefited for nutrients and materials transportation within the tissue after binding cells and scaffold. Zeolites dramatically improve samples porosity that is ideal for growth of dermal fibroblasts and cellular attachment.^92^ Furthermore, zeolite composites exhibited controlled swelling and degradation rate with proper microenvironment for wound healing. Oxygen molecules trap in zeolite pores during immersing because of the Vander Waals forces and oxygen molecules diameter is around 1.21Ǻ and Faujasite pores diameter is around 7Ǻ. Trapped oxygen is sufficient for dermal fibroblast activity and proliferation.^92^ It was reported that the copper‐activated faujasite/gelatin‐based wound dressing exhibited antimicrobial properties. Zeolites presence in the composites structure exhibited highest cell viability on NIH 3T3 fibroblasts, which was mainly related to the highly porous architecture (which can be regulated using zeolite) and its ability to enhance oxygen supply to cells^93^ contact angle was enhanced by copper activated faujasite addition, which may be caused by the interaction between the positively charged amino acids of gelatin with negatively charged zeolite particles, leading to the reorientation of the hydrophobic amino acid chains, mainly leucine, isoleucine, valine, phenylalanine, and methionine.^94^ After a short period of time, the contact angle was reduced because of water absorption, which then permeated into the pores of scaffold. The surface energy was also decreased for the samples due to the electrostatic interactions happened between gelatin and copper induced zeolite. These actions were done because if the surface become extremely hydrophilic, the scaffold would lack its integrity during its introduction to the culture medium. But then, a highly hydrophobic surface will hinder the adhesion of cells. So zeolites improve mechanical strength of the scaffolds but its content in the matrix should be optimized in order to regulate its absorption and nutrients diffusion rate and cell attachment.^93^

Salehi et al synthesized thermoplastic hydrogels based on zeolite nanoparticles/starch where herbal drug (chamomile extract) was encapsulated inside composites. Sustained drug release behavior was observed for zeolite‐embedded wound dressing composites with proper antioxidant and antibacterial properties (eg, chamomile exerts anti‐inflammatory effects through inhibition of lipopolysaccharide‐induced prostaglandin E2 release and attenuation of cyclooxygenase [COX‐2] enzyme activity without affecting the constitutive form, COX‐1). MMT assay revealed that zeolite samples have good biocompatibility especially for mouse fibroblast. Histological analysis on treated tissue samples showed enhancement in collagen generation, epithelialization, and inflammation reduction beside angiogenesis phenomena. These dressing displayed a better healing process for bed sore, ulcers, and burn wounds because of the modulated rates of fluid absorption, their porosity, and nanosized structure. It's noteworthy that the refractory ulcers of five patients were healed without any hypersensitivity reaction at clinical pilot study.^95^

Nitric oxide (NO) has a great effect on the regulation of biological processes such as vascular tone, neurotransmission, inflammatory cell responsiveness, defense against pathogens, and wound healing.^96^ Neidrauer et al examined a surface applied ointment containing nitric oxide‐embedded zeolite to evaluate the wound healing process. Satisfying results were observed for wound bacteria elimination and antimicrobial properties, namely, removal of gram negative and gram‐positive bacteria like *Escherichia coli*, *Acinetobacter*, *Staphylococcus*, and methicillin‐resistant staphylococcus aurous; beside its possess antifungal characteristics, as well. Analysis showed that zeolites presence lead to better distribution of components and enhanced adsorption of active agents’ due to their high surface area and porous structure. Embedding nitric‐oxide (NO) using zeolite resulted in controlled release and NO‐zeolite ointment exhibited proper wound healing with proper biocompatibility.^97^ It is known that the NO exhibits a critical function in the cutaneous response to UV radiation and in cutaneous inflammation. NO‐zeolite caused little inflammation, which induces a dermal CD4‐positive T‐cell infiltrate and IFN‐γ secretion. However, acidified nitrite induces an extreme epidermal infiltrate of macrophages with a same dermal infiltrate of CD3‐, CD4‐, CD8‐, and CD68‐positive cells and neutrophils. NO‐zeolite‐treated skin evaluation reveals the presence of the IFN‐γ and absence of the IL‐4.^98^ Liu et al fabricated a bandage based on polylactic acid fibers loaded with NO‐zeolite, which can be used potentially for diabetic patients.^99^

**FIGURE 6:**
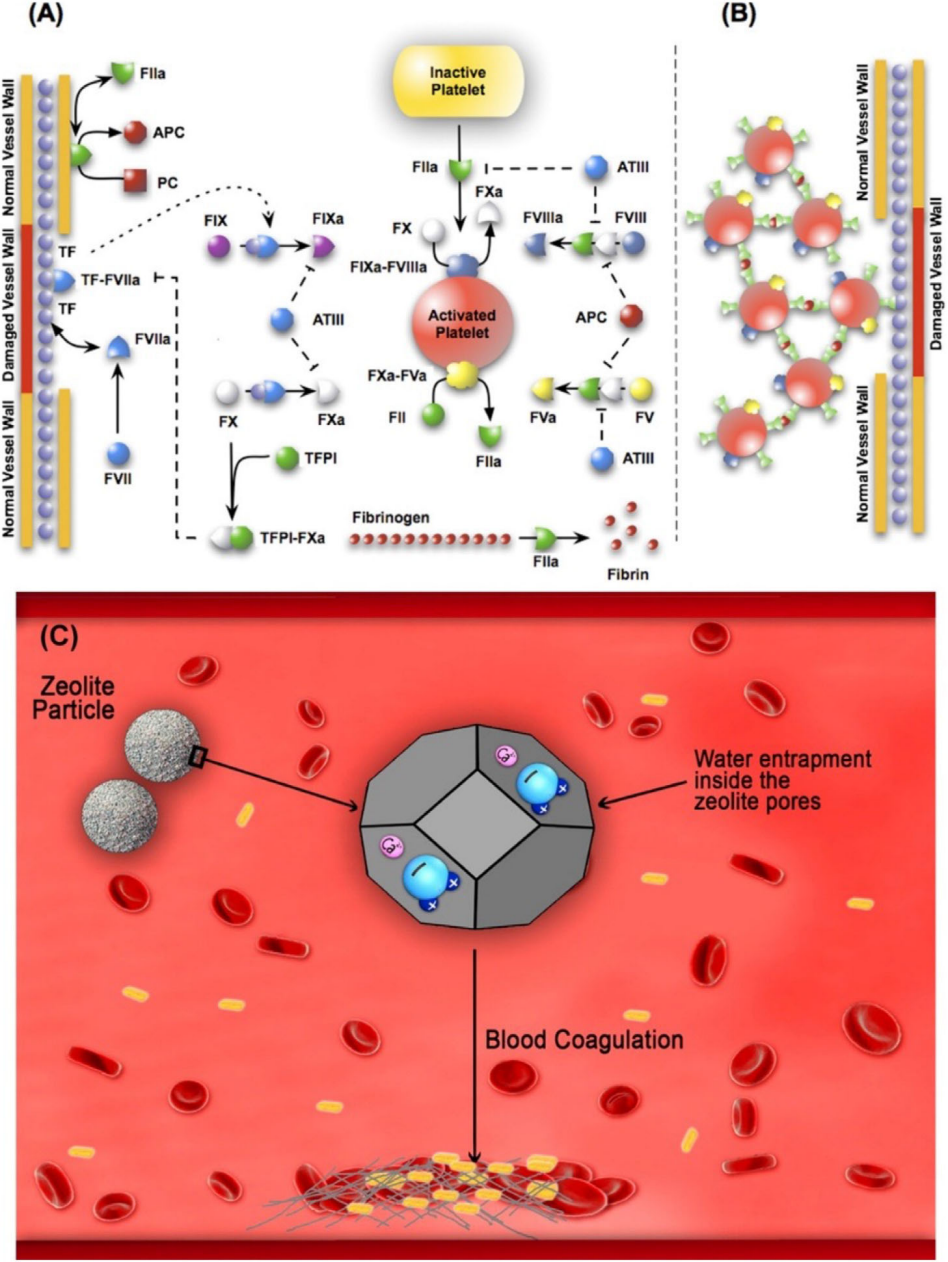
Schematic of the extrinsic coagulation cascade. A, Upstream coagulation factors are activated by substances exposed by vessel injury; chief among these factors is TF. Activated upstream coagulation factors initiate a cascade of events that culminate in the activation of platelets and the key protease FIIa. Thrombin forms an amplification loop by activating itself and other coagulations factors as well as platelets. B, Activated platelets then aggregate to form platelet plugs, which serve as scaffolds for fibrin clots. Summary of the potential hemostatic effects of zeolite. This schematic captures the effects of zeolite on the blood coagulation through absorption of water molecules into its pores resulting from the interaction with Ca2^+^ residing into zeolite pores. The interaction leads to concentrating the blood cells and clotting factors and promoting hemostasis. Adopted with permission^105^

Control of hemorrhage in its early stage prevents a great number of postinjury complications. Uncontrolled blood loss that occasionally occurs in ordinary or battlefield traumas is one the critical reasons of life loss in humans. For example, statistically, in severe war traumas 90% of the patients die before getting into any medical care facilities. So, an appropriate strategy for prevention of bleeding and infection seems to be necessary. Providing the wound with a dressing or a hemostatic agent are the most common pathways used normally in civilian or military situation. Zeolites are one of the most famous families of inorganic materials that have applied in these applications. Overall, hemostasis takes place via the synchronized action of three mechanisms: (a) vasoconstriction; (b) formation of a platelet plug; and (c) blood clotting.^100^ These inorganic hemostatic agents play role based on these major mechanisms: (a) absorbing water from the blood and concentrating the blood components at the hemorrhagic site; (b) activating the blood coagulation cascade; (c) providing a physical barrier to blood flow.^101^, ^102^ An effective design of zeolite‐based hemostatic agent must provide a fast, long‐lasting, and safe treatment of bleeding. The platform requires an appropriate selection of zeolite with a porous network enabling fast diffusion and adsorption of adsorbate molecules, and the zeolite should be immobilized that lets a long‐lasting usage and avoids the leaching. Moreover, zeolite‐based platform should be a nontoxic and low‐cost hemostatic system with easy applying method.^103^ Zeolites possess cage‐like cavities that can accommodate both water molecules and a variety of positively charged ions such as Ca^2+^ and sodium (Na^+^). The cations are relatively loosely held, so that they can exchange with other cations in contact with physiological solutions. Zeolites can entrap large volumes of water within their pores due to the electrostatic interaction between water and the Ca^2+^ that reside in open porous internal space. As a result, they concentrate coagulation factors and platelets in the hemorrhaging blood (Figure 6).^104^

**FIGURE 7:**
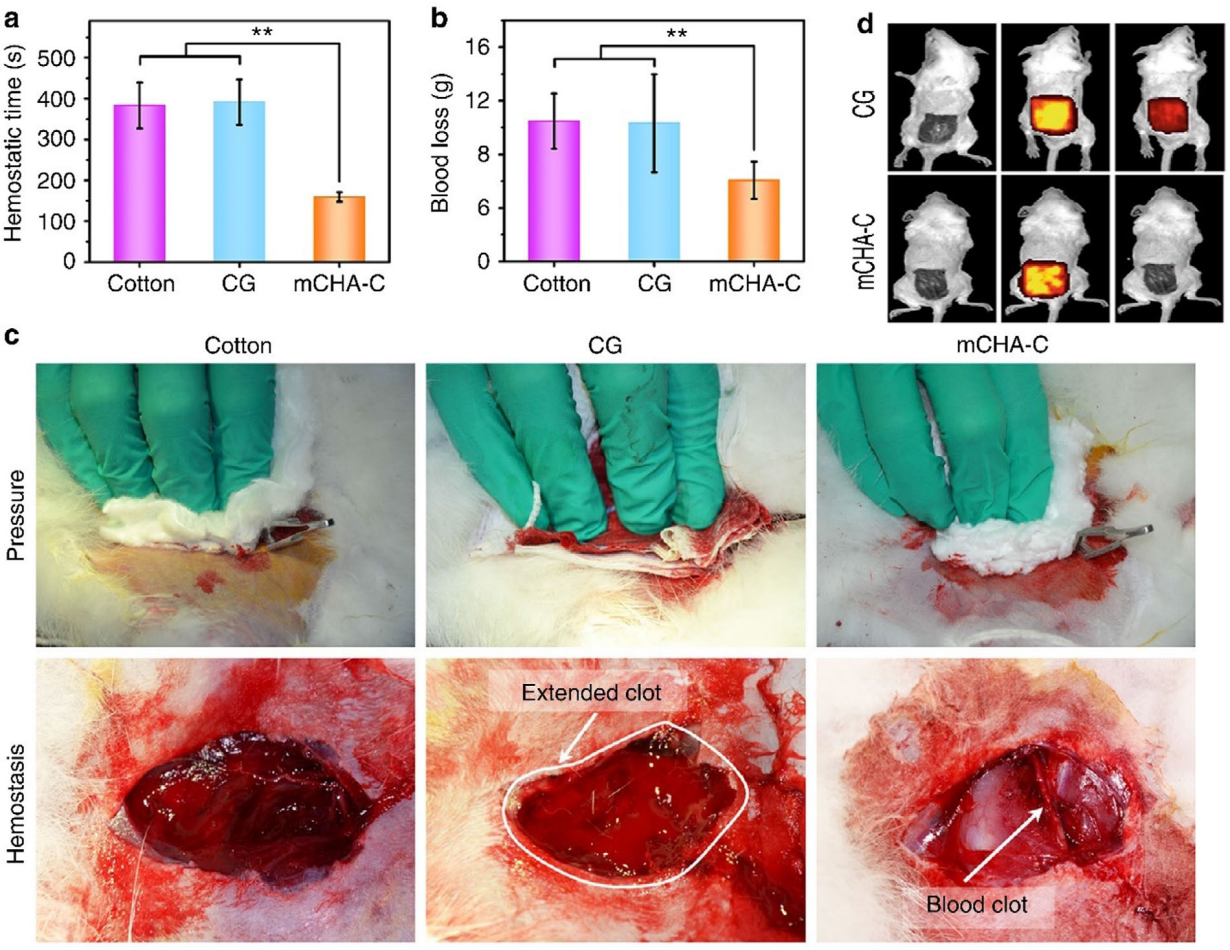
Comparison of pressure, combat gauze (CG), and zeolite‐cotton. Adopted with permission^103^

For instance, an injury simulated in femoral artery and proximal thigh in a swine was investigated by Alam et al who reported good outcomes after using a zeolite hemostatic dressing. Bio analysis tests showed that 1% zeolite dressing reduced the blood loss and mortality down to almost 0%. Resuscitation outcomes displayed that blood pressure and cardiac condition along with clearance of lactic acidosis were enhanced, specifically in the samples in which created wound was covered with a hemostatic dressing. Using standard wound dressings and bandages resulted in reduction of blood loss, but none of these dressings were able to stop the bleeding totally. On the other hand, injury samples treated with zeolite wound hemostats interestingly showed no continuous blood loss and bleeding begins to stop 3 min after its application on wound media. Dried zeolites fluid intake capacity effectively modulates the moisture and water content of the injury beside their good infection protection properties. Furthermore, the porous structure and high surface area of zeolites form a culture that antibacterial agents and ions can be easily put inside pores and be released during their implantation.^106^ Similarly, alginate/zeolite composite was fabricated to evaluate its capability for healing of a lethal groin injury with hemostatic properties. Practical animal simulation of incident exhibited that silver‐ and zinc‐treated zeolite and alginate composites dramatically settled the rate of mortality compared to untreated samples. Application of the compound zeolite hemostat can effectively control hemorrhage and dramatically reduce mortality from a lethal groin wound. Beside its biocidal features, Ag‐ and Zn‐substituted zeolite hemostats declined the exothermic reaction and attenuate the heat‐induced tissue injury.^107^ Zeolites can be synthesized in different shapes with adjustable properties. Li et al evaluated zeolites as a quick hemostats and healing agents in groin injuries. Zeolite‐based dressings were applied into a surgical wound in which their clotting degree, bleeding rate, post exothermic reactions, and scar formation were evaluated. In both conditions where a dressing is applied, no wound remained open after utilization of hemostatic agent and a complete clotting occurred in both wounds. The mortality rate dramatically decreased in zeolite granules dressing compared to 52% mortality rate of quick clot dressing used similarly. Necrosis of cells were observed in quick clot wounds tissues after healing procedure, whereas no sign of necrotic tissue cells was found in granulized zeolite‐treated wounds. Zeolite‐treated wound facilitated the inflammatory and proliferation phase causing proper wound closure. The zeolite cations hydration process in its framework is responsible for temperature elevation degree after its implantation at the moment of its mixture with water. The temperature increase after the exothermic reaction for zeolite revealed to be surprisingly low, which is attributed to the amount of Ca^2+^ content (near 1.93%) in comparison with Quick clot dressing, in which the calcium cations presence founded to be more abundant (11.43%). The lower contents of Ca^2+^ weakened the sudden elevation of temperature in zeolite samples and the side effect connected with the tissue burning.^108^ Yu et al fabricated flexible and tightly‐bonded mesoporous zeolite (Chabazite)‐cotton hybrid hemostat platform. The platform exhibited the high pro‐coagulant activity after water flow treatment (Figure 7). Chabazite zeolites possesses high dehydration activity that can play an efficient role in blood concentration and hemostasis. It is assumed that the great hemostatic feature of zeolites is related to the high microporosity and the large surface area.^103^

Esophagus ulcers are a sort of wound that mostly caused by swallowing very acidic or alkaline nutrients or chemicals. They are sometimes known as corrosive esophageal burn where the inflammation phase expands as a result of corrosive esophagitis prolonged presence. Eating or drinking strong corrosive materials, especially alkaline ones, may lead to severe perforation or even death. It was reported that platelet‐rich plasma (PRP) and Thymoquinone (TQ) had positive impacts on corrosive esophagitis both biochemically and histopathologically. It was deducted that the anti‐inflammatory effects and antioxidant properties developed by zeolites incorporated into culture of esophagus. TQ and PRP composites with zeolite showed positive outputs on recovery in esophagitis by lessoning inflammation in the contacted area.^109^ Moreover, proper oxygen supply to cells is a challenging issue in tissue engineering scaffolds. Ninan et al fabricate scaffold based on gelatin/copper‐faujasites (CAF) as a wound dressing. The dissolved oxygen measurements revealed that CAF embedded in the scaffold enabled enhancement of oxygen supply, which caused the promotion of cell proliferation. Animal model experiments displayed the ability of scaffold to promote skin regeneration.^110^

### 4.2 Bone tissue engineering

**FIGURE 8:**
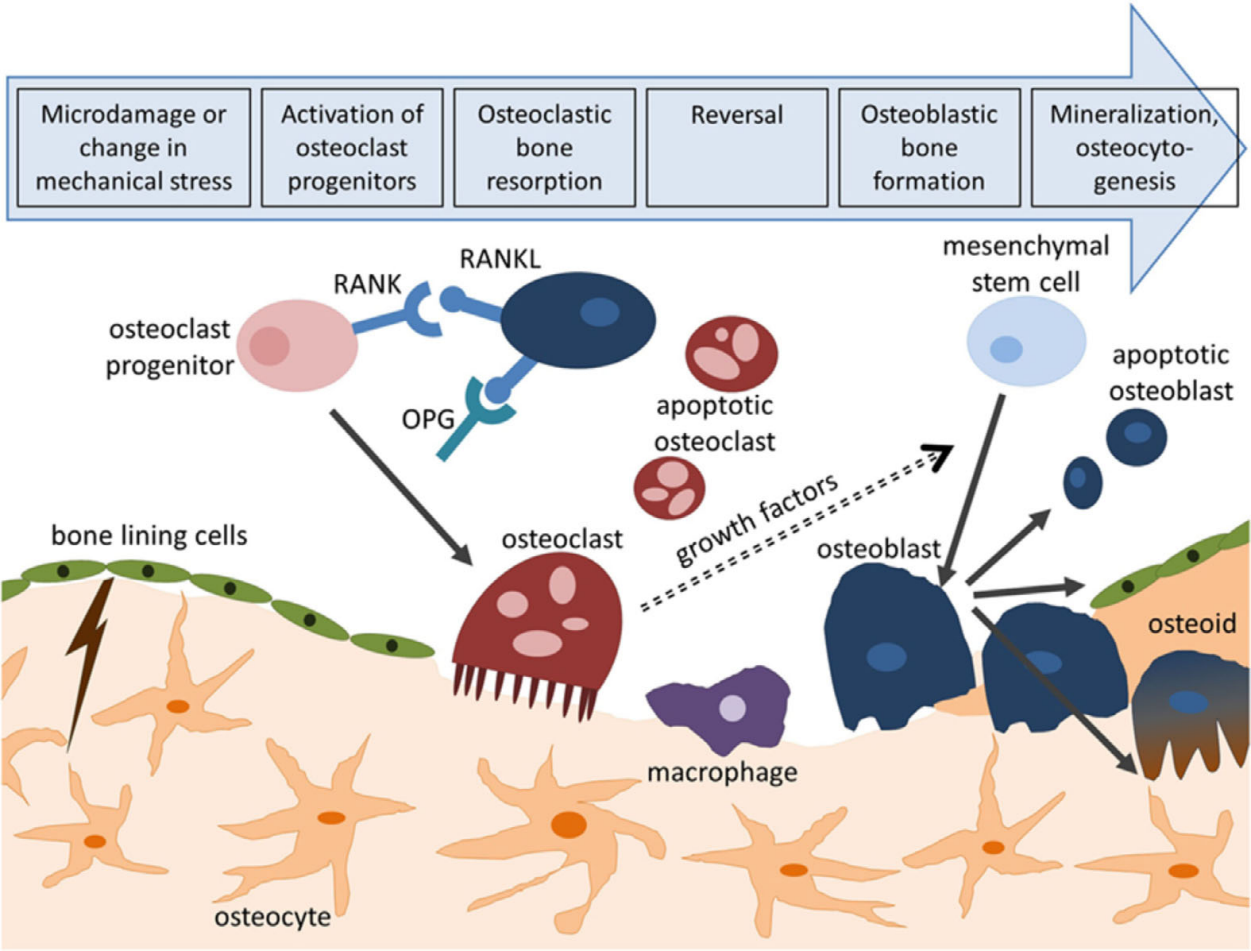
Bone remodeling process. Adopted with permission^112^

Bone in our body is a dual origin organ that is consisted of both inorganic and organic tissue materials such as collagen, fibrous elastin, and minerals. When this organ suffers any harm, bone tissue has the capability to simultaneously repair itself and start bone tissue regeneration procedure. Bone grafts such as autografts, allografts, and xenografts are routinely applied to fix bone tissues. Matrix and osteoclasts resorption are happened at the initial stages of the bone remodeling. Mononuclear cells provide the place for osteoblast at resorption site in which osteoblast generates the new matrix. In final stage of the bone remolding, matrix is mineralized, and some osteoblasts differentiate to osteocyte (Figure 8).^111^

However, their implementation into human body may cause some complications, namely, trauma impact or immune responses. Lately, biomaterial scaffolds and biological factors have created a new pathway toward bone regeneration by tissue engineering, which would interact directly with cells. Zeolites as inorganic aluminosilicate that possess good porosity and have proportionally large surface area^113^ with near zero cytotoxicity can assist in the process of efficient grafts and coatings synthesis, because the implants and scaffolds are suitable as a primary framework to keep the cells from defects and to improve the cell growth and proliferation.^114^, ^115^

Calcium‐attached zeolite/poly (amino acid) (CaY/PAA) composites with hemostatic properties were synthesized according to in situ melting polymerization route for bone implant applications. In this research, zeolite loaded with Ca was obtained from faujasite‐type zeolite NaY by ion‐exchange chemical pathway.^116^ Results indicate that the measured compressive strength of the CaY/PAA composites lie mostly between 145 and 186 MPa, after zeolite incorporation; thus, it possess suitable mechanical strength for load‐enduring bone replacement. Furthermore, the porous structure of zeolites endows the final composites with tunable release profile and encapsulation point, which regulate the fluid adsorption properties. In addition, coagulation analysis displayed that the CaY/PAA composites have shorter clotting period and better performance for promoting blood coagulation than other composites studied and also enhanced hemostatic activity.^117^ Iqbal et al synthesized a bioactive compound based on zeolite/hydroxyapatite using a microwave‐assisted wet precipitation method. Nanostructured zeolite‐HA composites exhibited proper bioactivity and cell compatibility and accelerate the formation of dense layer in tissues.^118^ LTA‐type zeolite is one of the popular zeolite materials for bone surface modification in different applications. It was revealed that addition of acid‐treated zeolite A into the bone area would help to decrease the number of pits per osteoclast at 24 h after treatment efficiently. Also, cathepsin B enzyme activity was brought down. Number of pits per osteoclast after 48 h of Zeolite A introduction into the media was calculated, which similarly showed a considerable reduction. However, it was found that this effect would not last for cathepsin B enzyme where its reduction rate nearly stopped or moderated. Zeolite A structure includes a tetrahedral, cage‐like framework. In aqueous acidic solutions like the one applied in this research, the chemicals partially dissociate releasing Si (OH_4_) and Al^3+^. Silicic acid and/or Al cations solutions had not the capability to amplify the impact of zeolite A incorporation to bone samples. The results obtained here suggested that residual structure of zeolite A or a component sub configuration of zeolite A should be the main reason for the reduction of OC resorbing capacity.^119^ It was reported that treatment of homogeneous strains of normal human osteoblast‐like cells with ZA induced a dose‐dependent increase in DNA synthesis of control and in the proportion of cells in mitosis.^120^

Compatible hydrogels are another kind of materials that could potentially widely applied in biomedical applications especially tissue engineering because of their convincing specifications. For example, Pazarceviren et al fabricated PCL‐PEG‐PCL tri‐block copolymer composite with clinoptilolite zeolite for bone tissue engineering by solvent‐free powder compression/particulate leaching synthesis route. The incorporation of clinoptilolite improved the mechanical properties and bioactivity of the composite in comparison with the simple PCL‐PEG‐PCL composite without clinoptilolite, which were attributed to the well porosity of clinoptilolite zeolite and its nanosized cavities. Protein adsorption capacity of the zeolites depends on the pI of the proteins, chemical structure of the zeolites, and pH of the environment. Clinoptilolite (CLN) had basic species (OH ions) in a solution at pH7.4 and CLN and BSA have pI around pH4.6 and 5.4, respectively. Although zeta potentials of the CLN and protein were found to be similar at neutral pH, BSA adsorption results showed that CLN could adsorb BSA at pH7.4. CLN readily interacts with the environment due to its positively charged ions such as Mg^2+^, Ca^2+^, and K^+^, and it also has high ionic exchange ability at the surface due to its small Si/Al ratio.^121^ Micropores of approximately 1‐µm diameter were detected adjunct to macropores (>100 mm) showing interconnective porous structure of the scaffolds. Macropores in the scaffold framework caused the particles to have larger surface area for cell proliferation and tissue in‐growth. Moreover, microporous inter‐pore cavities between macropores supply better cellular adherence into the scaffolds, whereas it facilitate nutrient rate and waste elimination.^121^ Davarpanah et al utilized poly(lactic‐co‐glycolicacid) (PLGA) in combination with nano zeolite particles as a biomaterial for bone tissue engineering. Electrospinning technique was used to fabricate such composites because nanofibers produced by the electrospinning approach include several beneficial outcomes such as notably high surface‐to‐volume ratio and small inner fibrous pore size with manageable porosity as well as their capabilities for reaching satisfying physical and mechanical properties. Zeolites interconnected channels with a clearly porous structure helped the final composite to bind with the cells properly and it also facilitates nutrients and other chemicals transportation, as these mentioned parameters are the basic reference points for a successful fabricated tissue. Also, other morphological characteristics showed that the electrospun zeolite nanocomposites could simulate the natural bone configuration effectively.^122^ Apatite formation and adsorption into the media increased constantly in the first 3 weeks until it reaches an ultimate saturation point where apatite content maximized and stopped. Bioactivity profile of the experiment may be addressed to alumina silicate phases, which were observed in XRD pattern. They cause apatite formation accordingly. Furthermore, mechanical tests revealed that pure polymer samples showed more elastic behavior and higher tensile strength, whereas introduction of zeolite into the polymer films scaffolds decreases the tensile strength proportionally. However, it increases samples toughness and fracture resistance and makes it close to the properties of native bone tissue, because it fills the gaps in the polymer structure and its fibrous porous framework limits the elasticity of pure polymer. PLGA is hydrophobic polymers that usually have large contact angles with water that diminish its water adsorption capability limiting its applicability in tissue engineering applications. But, zeolites incorporation through this polymers leads to a considerable decrease in water contact angles due to highly hydrophilic properties of zeolites and their large water intake capacity.^123^, ^124^

Type (A) zeolite nano particles were synthesized inside chitosan polymeric scaffolds shaping a novel nanocomposite. Using this nano composite as a bone tissue support assisted to overcome the shortage of donors issue and lack of bio compatibility besides moderating the hazardous consequence of using auto grafts and allografts in most of other case reports.^124^ Human mesenchymal stem cell introduced to the tissue scaffolds and stem cells viability, cytotoxicity, and cell attachment properties of scaffolds were evaluated. Such scaffolds showed proper proliferation and cell migration characteristics because zeolites modify the surface properties of chitosan scaffolds. Zeolites form open pores and interconnected pore channels that enhance the cell migration and growth resulting in better vascularization and generation of fresh cells that eventually constitute a new tissue segment. Furthermore, chitosan surface lacks any porosity that hinders the adhesion process, whereas by adding porous zeolite nanoparticles into the scaffold, a uniform film‐like layer of porous surface was obtained and its compatible pore shape and size improve cell adhesion and delivery of nutrients and oxygen, as well. In addition, zeolites inherent well fluid adsorption capacity helped them for a better permeability and diffusion rate of nutrients especially cytokines inside scaffolds and extraction of waste chemicals from them by entrapment of chemical substances in their porous culture.^124^

**FIGURE 9:**
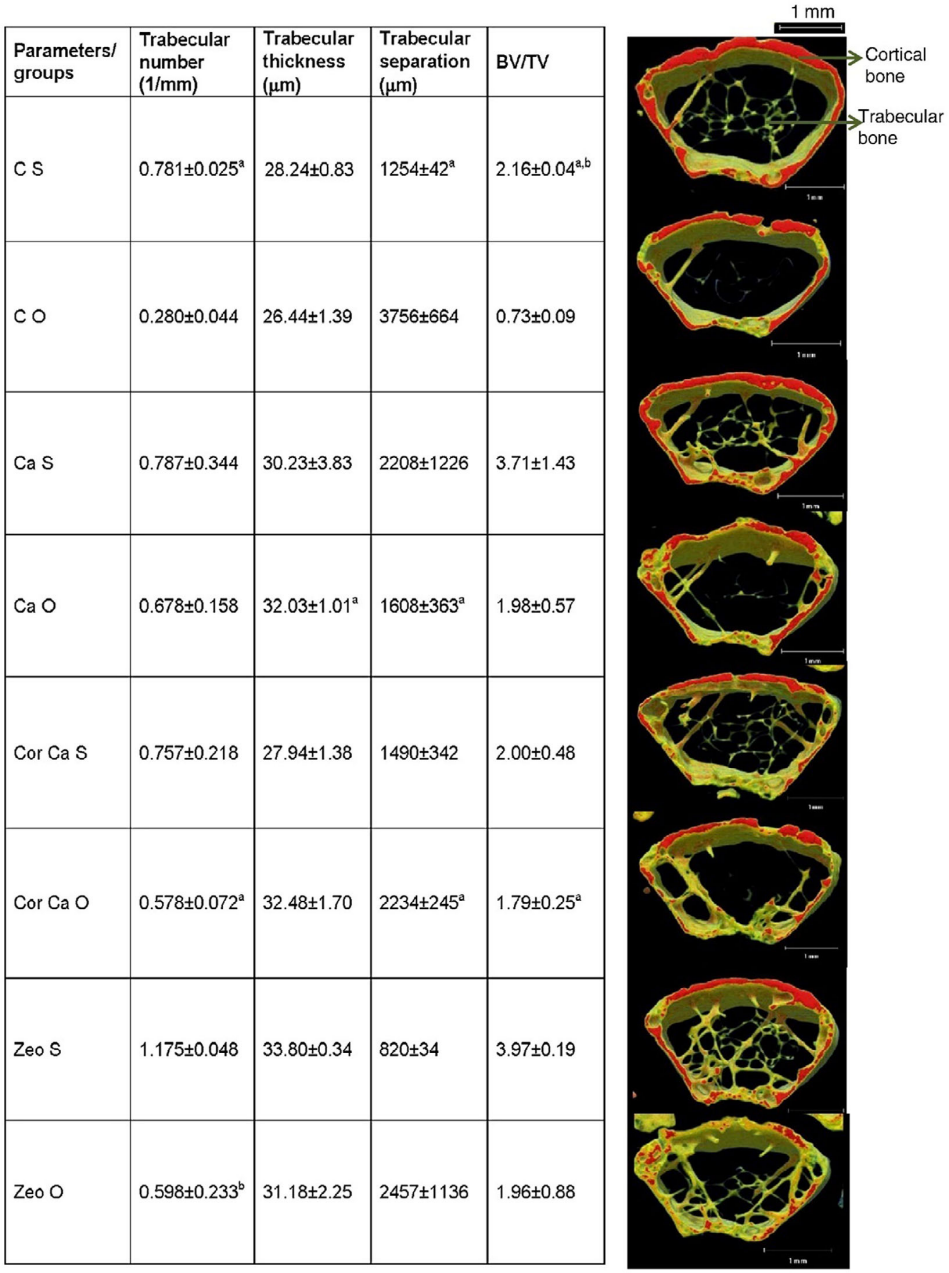
Effects of coral calcium and zeolite on the static histomorphometric bone parameters of the distal femoral metaphysis in ovariectomized mice on dietary treatment for 6 months Abbreviations: CS, control Sham; CO, control ovariectomized; CaS, calcium sham; CaO, calcium ovariectomized; CorCaS, coral calcium sham; CorCaO, coral calcium ovariectomized; ZeoS, zeolite sham; ZeoO, zeolite ovariectomized. Adopted with permission.^126^

In most of studies involving bone tissue engineering, total bone extract calcium deficiency founded to be one of the most vital parameters that jeopardize bone implants after implantations success. In osteoporosis condition, patients suffer from sever lack of vitamin D and calcium in their system that introduces them as potential candidates of fracture in different areas.^125^ So, supplementation of these people with adequate dose of calcium is significantly needed. In a research conducted by Banu et al, zeolites effect on protection of bone loss and coral calcium delivery was investigated. Different morphological and biochemical tests were performed to measure zeolites effectiveness toward bone regeneration and calcium content increase in bone area. Obtained results confirmed the benefits of zeolites in calcium ion delivery and bone growth. Pro‐inflammatory cytokines analysis indicated that zeolites incorporation notably reduced the ovariectomy‐induced increase, in TNF‐α, in comparison with numbers calculated for control mice. µCT densitometry analysis exhibited that trabecular number, trabecular thickness, and BV/TV all improve by zeolite powder samples addition into nutrient dietary blend after a specific time of surgery. Trabecular number increased to 1.17 (1/mm) in zeolite‐containing samples, whereas the most number that other samples reach in calcium and control samples is 0.78 (1/mm). Also, zeolites slightly increase the thickness of shaped trabecular bones subsequently (Figure 9).^126^ Hydroxyapatite‐based scaffold mimics the bone behavior because hydroxyapatite is the main component of the bone. Bedi et al used hydroxyapatite/zeolite to coat the titanium implant. Such coating enhances the osteoinduction (significant increment in BMP‐2 gene expression) and osteoconduction resulting in proper osteointegration, which facilitated the recovery and healing process.^127^ Moreover, it was reported that the MFI coating on titanium exhibited proper biocompatibility and enhanced osteogenic differentiation and bone regeneration.^102^ BMP‐2 as a ECM protein secreting by osteoblasts induces osteogenic differentiation of bone cell precursors.^128^ A more hydrophilic surface is widely accepted to provide better cellular adhesion for osteoblasts during bone formation, and crystalline hydroxyapatite surface is desired for faster osteoblast proliferation and differentiation.^129^, ^130^ Bedi et al coated the titanium with zeolite MFI‐hydroxyapatite as a bone implant. MFI enhances the implant hydrophilicity, which plays an important in the bone‐healing process. Zeolite topology delivers a high surface area for cell adhesion, and nano‐hydroxyapatite crystals offer anchor points for the extracellular matrix and the mineral secreted by osteoblasts during bone formation. Moreover, such coating compensates the elastic mismatch between titanium and bone, which enhanced the osteointegration and prevented the bone crack.^127^ It was reported that the zeolite MFI enhanced the osteoconduction, osteoinduction, and osteointegration. Promoted osteoconduction, osteointegration, and osteoinduction were observed in MFI‐coated implants, which are related to the 3D microstructure of zeolite crystals, and a microcrystal topology, which is proper for bone cell adhesion, proliferation, and growth. The bone formation process by osteoblasts necessitates cell adhesion to the ECM surface as an essential first stage to initiate proliferation and cellular differentiation, and to enhance deposition of newly synthesized ECM by mature osteoblasts. Because mineralization succeeds cellular adhesion and formation of organic ECM in osteogenesis, surface texture indirectly plays an important role in successful mineralization. The 3D surface offers attachment sites for colonizing immature osteoblasts, which in turn secrete ECM proteins for further anchoring and osteogenesis stimulation. Hence, MFI‐coated titanium implants may be more osteointegrative than bare implants. Moreover, such implant exhibited the proper anticorrosive feature.^131^

**FIGURE 10:**
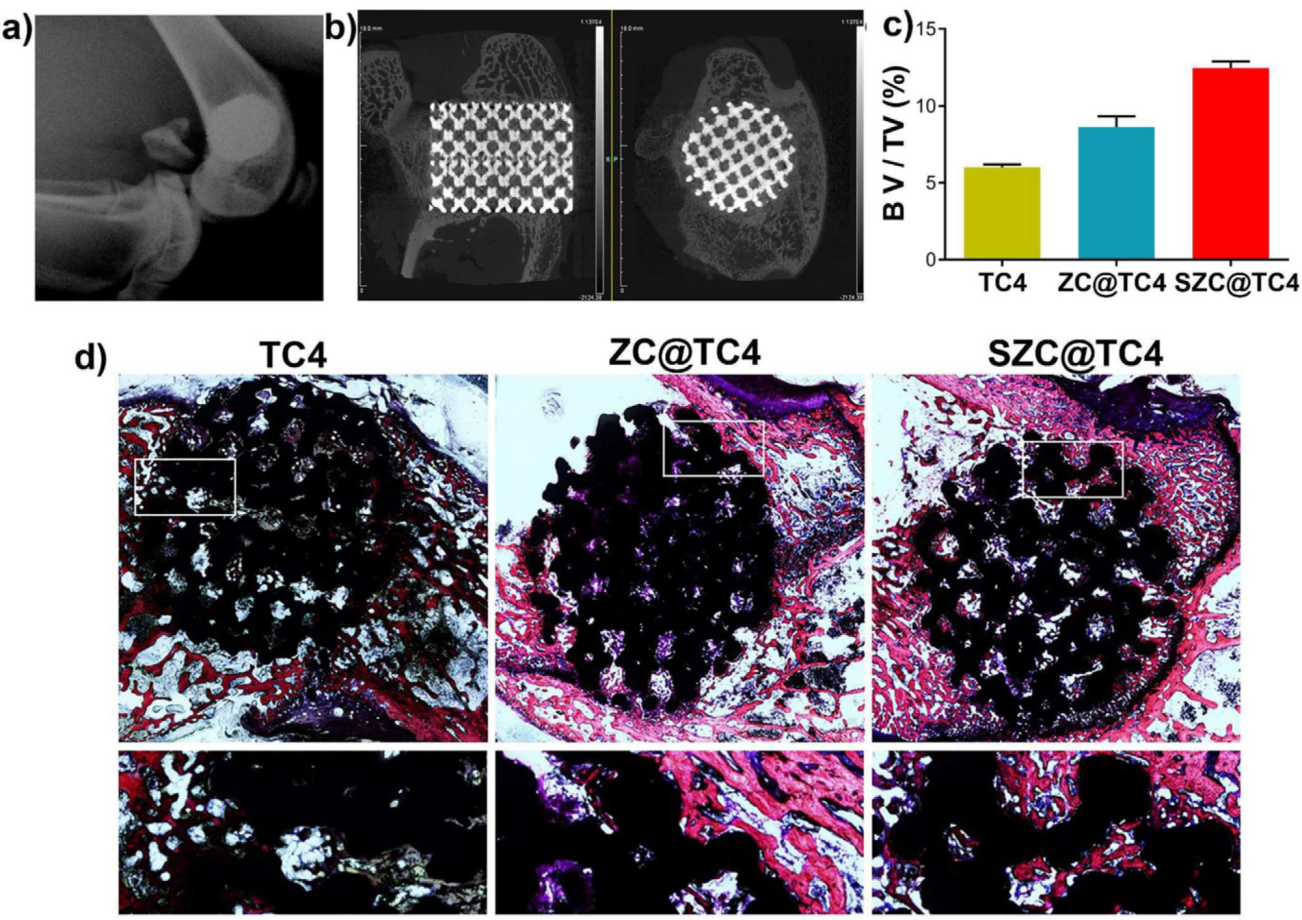
A, X‐ray of three‐dimensional printed TC4. B, Micro CT images. C, Quantitative micro‐CT analysis: Percentages of regenerated bone volume/total volume (BV/TV) in these implants; D, Van‐Gieson staining of histological sections Abbreviations: SZC, strontium ions incorporated zeolite coatings; ZC, zeolite coating. Reprinted with permission.^132^

In some cases, several cations can be incorporated inside porous frameworks for tissue engineering purposes. For example, Wang et al coated the bioactive alloy implants based on titanium with the strontium ions encapsulated within type A zeolite by in situ hydrothermal crystallization route. The obtained promising results suggested that zeolites could have great potential for orthopedic applications. Morphological analysis using SEM revealed that a bioactive coating with thickness of 1.3 µm has been tightly adherent along the sample, which also stably presents on rod braces of the 3D structure, indicating that a uniform bioactive coating all over the titanium alloy 3D structure has been shaped. The measured adhesive strength between zeolite coating and TC4 substrate founded to be near 6‐7 N. This value for adhesive strength for zeolite coating and substrate is much higher than previously studied coatings, such as hydroxyapatite and TiO_2_ fabricated by sol‐gel method or electrochemical process. The water contact angle for TC4 measured to be around 49°, whereas the contact angle for its zeolite containing composites significantly reduced to almost 9°; this may be mainly related to the hydroxyl functional groups and unsmooth texture of zeolite part of the composite. It should be considered that this angel mildly increases in the presence of strontium, which can be related to the weakly hydrated Sr ions that entered the pores of zeolite. Considering all of those positive points, zeolite‐incorporated samples after implantation also showed better cytocompatibility and less cytotoxicity in comparison with pure TC4 (Figure 10).^132^

Alloys play a crucial role in implants modification processes, which prepare the final materials for in vitro applications. Titanium alloy is one of the most common widely used materials in dental and orthopedic implants. Although it presented appropriate biocompatibility in numerous analyses, several reports showed that they could spread toxic ions into the tissue environment after implantation. Moreover, the elastic modulus of titanium alloys founded to be near 110 GPa that is significantly higher than bone range numbers (10‐40 GPa), causing a modulus mismatch and implant loosening, ultimately.^133^ A uniform, strongly adhered coating with high corrosion resistance was generated on the titanium‐based implant. The high corrosion resistance of MFI zeolite coatings makes them perfect choices for toxic aluminum and vanadium ions removal from alloy implants area into the tissue environment. Putting titanium within the zeolite framework provides a perfect adhesion to the substrates and can inhibit implant loosening, which are considered as very pleasing quality for bio implants. Zeolite coatings on titanium implants decrease the modulus mismatch with bone tissue, and have the capability to enhance osteointegration of implants, simultaneously.^133^ PVA/Col containing 5 wt.% zeolite exhibited the highest tensile strength of 2.34 MPa and its Young modulus is measured to be 11 MPa. These values suggest that zeolite (nano zeolite; average diameter, 20‐90 nm; specific surface area, 110 m^2^/g; density, 3.0 g/cm^3^) addition nearly doubled both the tensile strength and Young modulus of the sample. These results interpreted to be mostly due to hydrogen bonds between materials after zeolites addition into PVA. Collagen fibrous samples have larger angel than neat electrospun PVA. This may be the result of its peptide strands. A detectable reduction was observed in silica‐added composites due to the presence of hydrophilic silica nanoparticles, which have numerous silanol groups inside, on surface of fibrous material. In the zeolite nanostructures, substitution of Si (IV) for Al (III) in the framework induces a negative charge, which is consequently neutralized by an electrochemical equivalent of cations. The regulated distribution of negative framework and positive cations leads to a strong electrostatic field on the zeolite surface, which directly influences the interaction energies in polar adsorbents.^134^ Cartilage got especial attention during recent years in tissue engineering research. For example, Mehrasa et al examined electrospun‐fabricated polyvinyl alcohol (PVA) and collagen composite scaffolds connected with zeolite and silica nanoparticles. DAPI staining analysis showed that the presence of zeolite and silica in PVA/collagen composite notably increases the cell proliferation in these samples, respectively.^134^

### 4.3 Tooth engineering

As it is discussed, zeolite has been used for bone regeneration. Zeolite as a mineral combination of Na, K, and Ca with porous and hydrophilic structure causes effective bonding between scaffold and tissue. Due to large surface area, adjustable porosity, and proper mechanical strength, zeolite is a good candidate for bone and tooth tissue engineering. Semi‐permanent antibacterial feature of it makes it a proper candidate for dental applications. Mohandesnezhad et al used zeolite‐hydroxyapatite/PCL‐PLA scaffold for dental tissue engineering, which exhibited the proper biocompatibility and cellular activity.^135^ Zeolite has been used as an efficient canal irrigation fluid because of its unique properties. Ghivari et al injected silver‐activated zeolite solutions for *faecalis*, *Staphylococcus*, and *Candida albicans* bacteria elimination, which were more frequently found in dental media and caused most of root cultures failure after treatment. In such experiment, root canals failure was analyzed after zeolite solutions injection spatiotemporally. It was found that antibacterial behavior of solution increased by increasing zeolite concentration and its contact time, which would allow more entrapped silver cations and antioxidants reach canals surface that subsequently reduce bacteria concentration and canals failure.^136^, ^137^ Nikawa et al utilized silver‐incorporated zeolites in tissue conditioners in order to inhibit plaques generation on dental environment and prevent *C. albicans* formation. Tissue conditioners that have silver in their systems are suggested to possess prevention and removal attributes for different bacteria in saliva immersed solutions up to 4 weeks. This beneficial act is related to the homogenous nanopore distribution of zeolites, which let them to transport larger amounts of antibacterial agents into the solution, and their slow release behavior perfectly regulates chemicals entrance into the environment that guarantees a constant concentration of silver antibacterial agent in long period.^138^ Bedi et al utilized Ag‐doped zeolite as an antibacterial surface exhibiting the long‐term antibacterial activity and proper stability.^139^ Zeolite because of the changing the surface charges prevents the fouling formation and bacterial attachment.^140^ Ag^+^‐exchanged zeolite (clinoptilolite) exhibited better performance than Zn^+^‐exchanged and Cu^+^‐exchanged zeolite. Ion‐exchange isotherms exhibited high selectivity of Ag^+^ over Na^+^, and fully exchanged with Na^+^.^141^ Metal ions presence enhanced the Ag^+^‐zeolite activity because of enhancing the Ag^+^ exchange out of the zeolite into solution.^142^ It has been reported that the concentration of 1‐10 µg/mL for Ag^+^ and 10‐100 µg/mL for AgNP is essential for antibacterial performance.

Polymer substances were widely used as dental prosthesis. One the most well‐known polymers applied is poly(methyl methacrylate) (PMMA) complexes. However, PMMA products have more vulnerability toward forming bacteria on them that predicted to cause post‐bacterial complications after its placement. Regarding these findings and deductions, introduction of antibacterial Ag‐zeolite that is a promising antimicrobial and antibacterial agent reduces the formation of bacterial species in dental culture. Zeolites provide numerous benefits in this application because its cytotoxicity is substantially low; besides, it can easily be bonded and mixed with polymer agents to modulate their release properties and shape antibacterial composites by bringing different functional groups inside the polymers to compensate for their weakness.^143^ Handmade dental base structure resins are also considered to be modified by zeolites in order to improve their cleanness and sterile condition because they will be put in direct contact with body tissues. Cosemiro et al studied the effect of zinc/silver blend zeolite nanoparticles on accumulation of bacterial and microbial agents in the denture base of resins prepared. The results revealed that they have an effective role in antimicrobial interactions against *Streptococcus mutans* and *C. albicans*. But, addition of zeolites in higher dosage may have negative impacts on samples mechanicals properties.^144^ Self‐cured acrylic resins have been attracted significant attention in dental regeneration realm, because such resins were used as temporary crowns and repairing fractured segments. These self‐cured acrylic resins are exposed to contaminations and they have a good chance of being affected by bacterial or microbial groups, which causes a very severe and annoying smell after its contamination. Analysis indicated that just a short window of time is needed for growth of bacteria in the resins among their exposure to *C. albicans* as a sample bacteria element. However, in samples mixed with silver contained zeolites it was observed that level of contamination and necessary time span for bacteria's growth decreased notably. Zeolites protection ability is associated with their porous surface and large number of micropores to encapsulate and transfer silver ions to resins surface in time. Furthermore, the mechanical properties of zeolites make it possible to create a film coating on the resins and prohibit contaminates.^145^, ^146^ Periodontics is another part of dental science that zeolites may have beneficial improvements in them. For example, zeolites were utilized in form of solution in PBS for washing dental or mouth area, which presented a reduction in supragingival plaque creation. This reduction was attributed to zeolites inherent antibacterial effect beside their high surface area and well porosity, which can selectively adsorb bacterial agents and generated plaques and protect the main tissues from any bacterial contamination or plaque formation.^147^, ^148^, ^149^

### 4.4 Hemodialysis

One treatment for kidney failure is called hemodialysis. Hemodialysis is process of filtering wastes and excess water out of blood, as healthy kidneys do. Hemodialysis helps to control blood pressure and balance important minerals, such as potassium, sodium, and calcium, in your blood. During treatment, your blood travels through tubes from your body into the dialysis machine. In the dialysis machines, the blood passes through a filter (ie, dialyzer) that removes waste and excess water. After that, the blood travels through tubes from the dialysis machine back into your body.^150^

**FIGURE 11:**
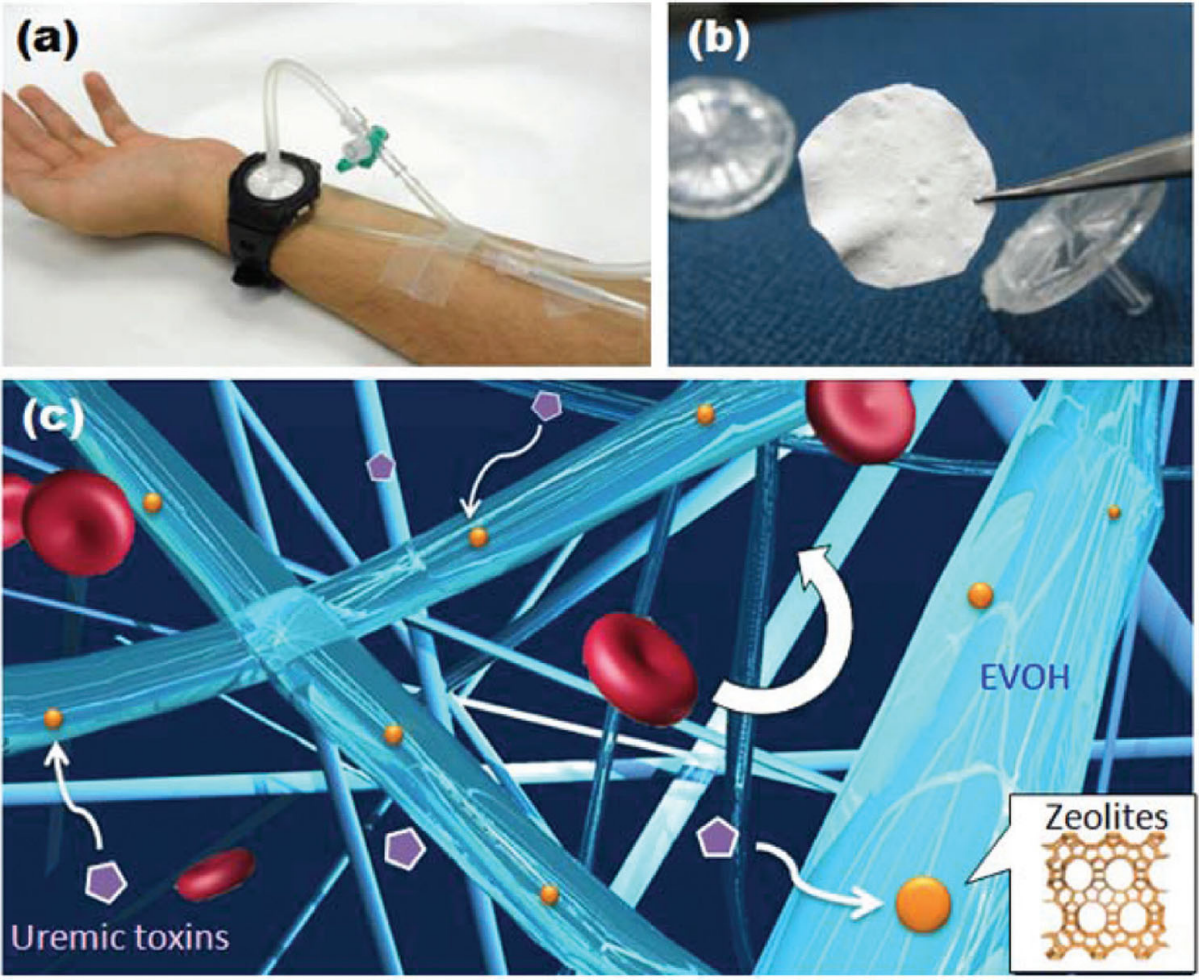
A, Wearable blood cleaning system. B, Zeolite composite nanofiber. C, The mat is based onpoly(ethylene‐co‐vinyl alcohol) as the based matrix/zeolites, which enable to remove uremic toxins. Reprinted with permissionm^151^

In applications of artificial kidneys, zeolites have been used as dialyzers. The selective absorption of zeolite is due to its crystalline structure, which is connected by channels and holes of a uniform size. A molecule that has the size and dimensions appropriate to the size of the canals or pores can enter and be absorbed into the internal cavity. Zeolite membranes were able to separate urea, uric acid, p‐cresol, creatinine, and indoxyl sulfate from hemodialysis fluids in a dialysis wheel system. In this way, hemodialysis fluid is able to be reused, in which some results are up to 10% better than conventional hemodialysis. Zeolite with polymer substrate was used to remove uremic venom and blood purification. Zeolites porosity helped the amorphous polymers to efficiently absorb toxins, and polymers’ layer by layer composite ability assisted the configurationally better adjustment of filters inside the place defined. Also, zeolites enhanced the mechanical ability of the samples toward tensions (Figure 11).^151^, ^152^, ^153^ Lu et al utilized polyethersulfone‐zeolite composite membrane to separate the indoxyl sulfate (an important toxin in causing reno‐cardiovascular syndrome). Electrostatic attraction was the main adsorption mechanism in zeolite membrane.^154^

### 4.5 Antimicrobial agents

Antibacterial activity is attractive issue in biomedical application. Hence, antibacterial surfaces have been developed. Zeolites because of large ion exchange feature are capable to be loaded with large number of ions. The incorporation of metal ions into zeolites increases antibacterial properties.^155^ Metal ions have been utilized as antimicrobial agents. Silver, zinc, and copper as the most famous metal ions have been used as an antibacterial agent. However, such ions simply oxidize and lose their antimicrobial properties because of change oxidation state from X^+^ to X^0^. Hence, a precise host is required such as zeolite to exchange ions with metal ions.^156^

**FIGURE 12:**
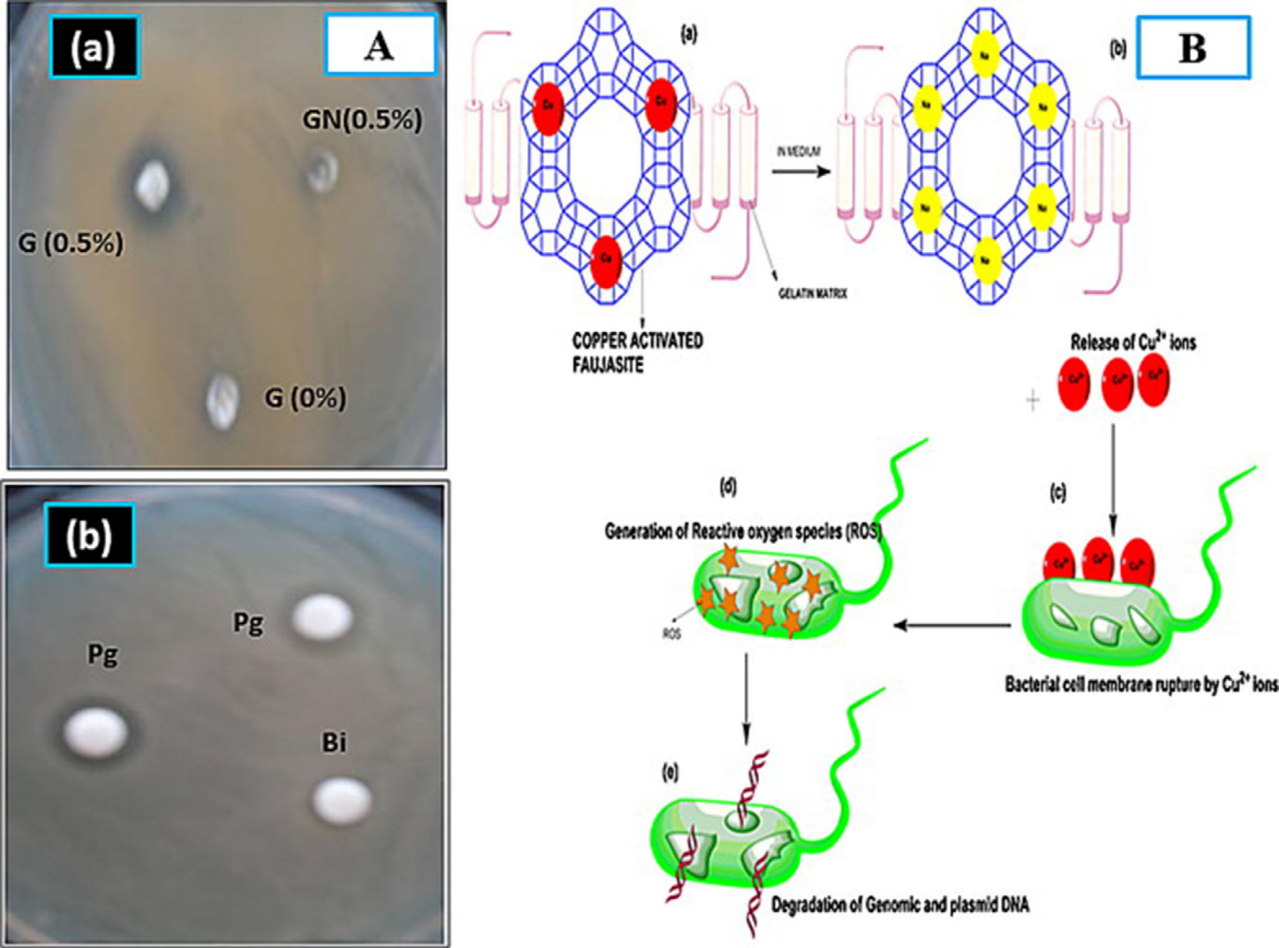
A, Agar disc diffusion tests of (a) G (0%), GN (0.5%), and G (0.5%); (b) positive control discs, Penicillin G and Bactopin against *Escherichia coli*. All tests were carried out in triplicates (n = 3). B, Schematic diagram showing bacterial lysis by copper released from pore of CAF. Reproduced with permission.^110^ G (0%) is the control without CAF. G (0.5%) is scaffolds with 0.5% CAF. GN (0.5%) is the scaffold with 0.5% (w/v) of faujasite without any copper

It was reported that porous magnesium‐x zeolite composite scaffolds with specific amounts of zeolite were synthesized using hybrid powder metallurgy and space holder fabrication techniques. Morphology tests revealed that zeolite interconnection to the scaffold had the minimum impact on its porosity and pore size distribution, which means that addition of zeolite had not tampered the effectiveness of the samples.^157^ Besides, the MTT assay study revealed that cell viability is higher for the Mg/zeolite composite scaffolds compared to the neat Mg scaffolds over the duration of the experiment. It was suggested that by increasing the amount of zeolite in samples surface, cell proliferation improves, which is mostly attributed to the high‐protein‐intake capacity of zeolite that originates from their silicate origin. As we know, zeolites have two basic substructures (SiO_4_)^4−^ and (AlO_4_)^5−^ in which both Si and Al chemical parts are mitogenic for bone cells. Al stimulates neo‐osteogenesis, whereas Si is an abundant material in most of bone cell growth media. This research clearly showed that encapsulation of Ag into zeolite in these composite scaffolds can offer relatively long‐term antibacterial activity for zeolite samples resulting in its efficient impact for bone infection prevention. Silver (Ag) is preferable metal for metal‐exchanged zeolites due to its excellent antibacterial activity.^157^ Other zeolites can also be used for improving antimicrobial and antibacterial properties of materials. For example, in a research study zeolite coating on titanium alloy surface had been fabricated in order to decrease the peri‐implant infections phenomena and enhance their antibacterial as well as anti‐adhesive capabilities. As a result, continuous zeolite (A) thin aluminosilicate films with a thickness of near 1 µm were coated in its surface through in situ crystallization route, which should be performed under hydrothermal conditions. Super hydrophilic aluminosilicate coatings generate an applicable pathway to drive back the bacteria accumulation. Utilization of inorganic substrates with a considerable hydrophilic property may assist the proliferation of cells while preventing bacterial growth. Low surface energy hydrophobic Medias bind with biomolecules, cells, and bacterial environments by electrostatic or van der Waals relations. Therefore, when they became hydrophilic, they would be covered with hydrophilic chemicals and strong hydrogen bonds like a thin film of water in the surface. Similarly, zeolites hydrophilic characteristics may improve antibacterial properties in the surface according to these interpretations.^158^ In another study Ueshige et al investigated the antimicrobial and viscoelastic properties of Ag‐zeolite composite for different tissue conditioners, namely, Visco gel (VG), GC Soft‐Liner (SL), FITT (FT), SR‐Ivoseal (IV), and Shofu Tissue Conditioner. The obtained results indicated that due to high porosity and abundant hydrogen bond cites in zeolite/silver composites surfaces, they exhibited good antimicrobial and antibacterial interactions during the experiments. On the other hand, mechanical and viscoelastic analysis showed that dynamic viscoelastic properties have not been altered in all tissue samples. Specifically, no change has been observed in tissue conditioners where some improvements have happened in other tissue samples. Viscoelastic tests were performed because they can closely simulate the oral conditions of a body and it provides us with useful information for oral administration. Oral condition specifications, which must be provided for accurate simulations, include 37°C (ie, physiological temperature) and frequency of 1 Hz. Ag‐zeolite composite predicted to have direct impact in the penetration rate of chemical materials known as plasticizers, and as a matter of fact, the inherent dynamic viscoelastic characteristics of the materials are estimated to be altered. But, the power to affect the properties differed in the materials consumed in this research experiments and this fact may be demonstrated by the different molecular weight of the plasticizers that regulate the effect of zeolite on them, which eventually leads to the different viscoelastic numbers achieved.^159^ Moreover, surface functionalization can increase the antibacterial activity. Hanim et al decorated the Ag‐zeolite NaY surface with ‐NH_2_ groups, which increased the antibacterial activity against a wide range of bacteria such as *E. coli* and *Staphylococcus aureus*.^160^ Ninan et al fabricate the CAF antibacterial and wound healing purpose. A mechanism of the antibacterial feature of the scaffold is depicted in Figure 12. When the scaffold was immersed in the medium, cation exchange occurred, and copper ions were released. The released positively charged copper ions interacted to the negatively charged cell membrane of bacteria via electrostatic interactions. Because of oxidative damage, cell membrane became permeable, and copper ions penetrate to the cell and damage the DNA by releasing reactive oxygen species (ROS) (Figure 12).^110^ Demirci et al investigate the antimicrobial properties of zeolite type X and A: with different Al/Si ratio, ion exchanged with Ag^+^, Zn^2+^, and Cu^2+^ ions. It was observed that Ag^+^ ion‐loaded zeolites exhibited more antibacterial activity. It was noticed that various synthetic zeolites can be ion exchanged with Ag^+^, Zn^2+^, and Cu^2+^ ions to achieve antimicrobial features with prolonged and stronger activity of ion‐releasing characteristics.^161^

Zinc silicate inorganic composites were synthesized via 3D printing and freeze‐drying method in which silver ions were encapsulated by ion exchange.^162^ The nano‐fibrous attapulgite that has appropriate rheological characteristics and good bioactivity is combined as an inorganic binder into the synthesis solution for bone tissue engineering purposes. The zeolites exhibited fundamental advantages in biomedical science; normally, zeolites modified with functional ions for tissue engineering are mostly consisted of aluminosilicate‐type inorganic materials, which inevitably release aluminum during their application that cause adverse effects for tissues and interfere with the mineral metabolism of target organism.^163^ However, zeolites have the benefit to be synthesized in the absence of aluminum source where another cation replaces with Al cation and form a facile metal silicate substance successfully. Hence, it is significantly crucial to synthesize aluminum‐free samples for preventing tissue contamination in certain applications. Zeolites and even these sorts of silicate materials possess good strength properties, which found to be one of the vital points for the regeneration of hard tissue samples; for this reason, the 3D‐printed scaffolds here may present desired mechanical strengths to be compatible with other sections in the growth process of the implanted tissue.^164^

The mechanical properties of standard human bone comparison with mechanical properties of silver‐coated zeolite in this experiment indicated that there are notable similarities between these bone samples, proving zeolites ability to efficiently replace bone tissue and be used in bone repair operations. The reinforcement mechanism of Ag‐3DPZS is largely related to the nano fiber structures of attapulgite as printing inks binder, which improves the viscosity and the plasticity in zeolite and metal silicate samples.^164^ Zeolite‐A/chitosan hybrid composites were fabricated successfully with desired properties in order to optimize its condition for bioapplications. The variable parameters include aluminosilicate contents and shapes, degree of porosity, and well mechanical strength. Morphological analysis revealed that incorporation of zeolites into the scaffold has provided the system with macropores with average pore size of 100‐300 µm. The compressive mechanical strength of the zeolite containing composite increased with zeolite content, getting to a notable amount of 3.2 MPa, which is accordingly 4.5‐fold higher than that of pure chitosan scaffold. The tests’ results exhibited that hydroxyapatite tended to grow faster in zeolites than pure scaffolds of chitosan where few hydroxyapatites were detected.^165^ Also, presence of calcium cations in crystal structures of zeolite helped the process of hydroxyapatite mineralization. The 35 wt.% silver‐zeolite A/chitosan composite displayed the highest antimicrobial activity. This mostly can be attributed to two main reasons; first, the chitosan matrix in the hybrid composites, which is positively charged, has the capability to bind with negatively charged *E. coli* cites by electrostatic interaction, leading to a better coverage of *E. coli* system with silver ions. However, this process has some obstacles, namely, low surface area of chitosan films and poor porosity that directs us to the second reason, that is, the high porous structure and good surface area of zeolites that can rectify the samples and enhance their ability to eliminate microbial agents efficiently.^166^ Table 1. summarizes the main zeolite‐based scaffold in tissue engineering.

**TABLE 1:** Zeolite application in tissue regeneration

Type of zeolite	Targeted tissue	Properties	Ref.
Gelatin/copper activated faujasites	Skin	Antibacterial properties and proper wound healing, enhanced the dissolve oxygen level	110
Zeolite A loaded with nitric oxide	Skin	Used as s a topical ointment, antibacterial effect, proper wound healing	97
Zeolite A/chitosan	Skin	Film properties can be adjusted by zeolite addition, adjusted swelling behavior	167
Zeolite A/Pectin	Skin	Proper wound healing, controlled drug delivery, transparency, good swelling properties, good oxygen transmission rate, and biocompatibility	168
MFI/Hydroxyapatite	Bone	Super‐hydrophilic and anticorrosion coating	127
Zeolite‐Y/Hydroxyapatite	Bone	Good bioactivity and proper biocompatibility	169
Zeolite/strontium	Bone	Anticorrosion coating, biocompatibility, increase the alkaline phosphatase (ALP) activity	132
MFI	Bone	Anticorrosion coating, biocompatibility, improved osteointegration	131
Zeolite/ Hydroxyapatite in PCL‐PLA nanofiber	Tooth	Enhanced cell adhesion, proper cell growth	135
Zeolite/Silver	Tooth	Antibacterial properties, restorative material, root‐canal filling material	170, 171
Zeolite A	Homeostasis agent	Blood clotting performance	172, 173, 174

## 5 CLINICAL AND PRECLINICAL STUDIES

To develop the zeolitic‐based material in medicine, the preclinical and clinical studies should be performed. Flower et al. performed the clinical study including 22 human subjects. The zeolite (clinoptilolite) impacts the chronic infections that could be tracked back to heavy metal contaminating. Through treatment with zeolite up to 30 days, urine and blood serum were gathered and analyzed for heavy metals and electrolytes. It was demonstrated that the regular use of zeolite was efficient in elimination of toxic heavy metals from the body through urine.^175^ Zhakov et al performed other clinical study about the detoxifying effect of zeolite to remove the heavy metal‐poisoned 102 men after 1 month. Reduction in heavy metal (Cd, Pb, Cu, Cr, and Ni) content was a consequence of the zeolite detoxification performance and possible renewal of the body mineral metabolism homeostasis.^176^ Pavelić et al performed a preclinical study to evaluate the zeolite toxicity. In this research, the impact of the exposing time was evaluated in three classes: (a) acute toxic responses in rats/mice model up to 1 month, (b) subchronic toxic responses in rats/mice model up to 3 months, and (c) chronic toxic responses up to 6 months in mice and 12 month in rats. There was no toxicity observed in groups.^177^ The oral administration of the zeolite in a clinical studies exhibited a positive effect on the intestinal tract as it positively influenced the intestinal wall integrity.^178^ Preclinical results showed a positive effect on the intestinal microbiome.^179^

It was observed that the zeolite administration in a controlled clinical trial stimulated a major increment in femoral bone mineral mass in osteoporotic women,^180^ which causes the morbidity and mortality worldwide. Direct correlation between silicon content and bone formation was shown.^181^ An evaluation on 3198 women (middle‐aged) displayed that silicon interacts with the oestrogen status on bone mineral density, suggesting that oestrogen status is important for the silicon metabolism in bone health.^182^ Zeolite powder suspended into phosphate‐buffered saline to prepare silver zeolite mouth rinse. Clinical study proved that silver zeolite considerably decreased plaque forming in comparison to the placebo^183^ silver‐exchanged zeolite as a tissue conditioner exhibited antimicrobial effect. Based on these findings and a double‐blind clinical study, the drug zeolite was registered for dental purposes.^148^ In a clinical trial, high silica zeolite showed a selective adsorption of the inhalation anesthetic desflurane in an anesthesia machine. Despite charcoal filters, complete desorption and condensation of desflurane to the liquid with high purity (about 85%) happen at moderate temperature using zeolite adsorbers.^184^

However, the number of clinical trials of zeolitic materials on human body is still low, and the zeolite effect on human body should be evaluated deeply.

## 6 CONCLUDING REMARKS AND FUTURE PERSPECTIVE

Zeolites are natural or synthetic materials with multifarious characteristics useful for tissue engineering. The microstructure of zeolite is tunable by controlling the pore size, pore morphology, and elemental composition that function as for different types of scaffolds. Zeolite appeared promising due to appropriate wound healing, blood coagulating, and antibacterial characters required in wound dressing and skin regeneration. Zeolite as a mineral has also been frequently used in bone and dental tissue engineering. In this review, we have endeavored to illuminate the zeolite properties and applications in tissue engineering to pave the way for appropriate scaffold design for tissues and organs. In this review, reports on the use of zeolite in tissue engineering and the main outcomes are classified:

- Skin and wound tissue engineering: It was revealed that zeolite because of hemostatic and antibacterial properties can play an important role in skin regeneration. Moreover, in wound dressing zeolite addition can be used for achieving desired mechanical properties and altering the water vapor transition rate.
- Bone/tooth tissue engineering: It was explored that zeolite because of the proper mechanical and biological properties can be utilized in bone/tooth tissue engineering.
- Antimicrobial agent usage: It was revealed that zeolite can be a good host for antibacterial agents that can be released in desired manner and site.

Zeolite combination with other biomaterials such as polysaccharides can be used as a scaffold with adjustable properties such as porosity and mechanical performance. Zeolite due to the unique properties is utilized in various applications from pollution removal to biomedical. Zeolite can encapsulate the drug and therapeutic agent and release in controlled manner. Moreover, due to ion‐exchange feature it can be used as a detoxifying agent. Moreover, zeolite has been used in imaging system that can be used as a diagnostic platform and along with therapeutic agents can be used as a theranostic device. Possibilities for designing zeolitic structures by the computational simulation methods enhance the zeolitic substrate performance. The use of such fundamental simulation/modeling computational approaches provides the engineers a general image on the zeolite structure required for a target tissue or organ repair. The clinical trials should be performed to widen the zeolite usage in medicine commercially.

## CONFLICT OF INTEREST

The authors declare no conflict of interest.
